# Assessment of Therapeutic Interventions and Lung Protective Ventilation in Patients With Moderate to Severe Acute Respiratory Distress Syndrome: A Systematic Review and Network Meta-analysis

**DOI:** 10.1001/jamanetworkopen.2019.8116

**Published:** 2019-07-31

**Authors:** Hiroko Aoyama, Kanji Uchida, Kazuyoshi Aoyama, Petros Pechlivanoglou, Marina Englesakis, Yoshitsugu Yamada, Eddy Fan

**Affiliations:** 1 Department of Anesthesiology, Graduate School of Medicine, University of Tokyo, Tokyo, Japan; 2 Interdepartmental Division of Critical Care Medicine, University of Toronto, Toronto, Ontario, Canada; 3 Department of Anesthesia and Pain Medicine, The Hospital for Sick Children, Toronto, Ontario, Canada; 4 Peter Gilgan Centre for Research and Learning, The Hospital for Sick Children Research Institute, Toronto, Ontario, Canada; 5 Institute for Health Policy, Management and Evaluation, University of Toronto, Toronto, Ontario, Canada; 6 Library and Information Services, University Health Network, Toronto, Ontario, Canada

## Abstract

**Importance:**

A number of interventions are available to manage patients with moderate to severe acute respiratory distress syndrome (ARDS). However, the associations of currently available ventilatory strategies and adjunctive therapies with mortality are uncertain.

**Objectives:**

To compare and rank different therapeutic strategies to identify the best intervention associated with a reduction in mortality in adult patients with moderate to severe ARDS.

**Data Sources:**

An electronic search of MEDLINE, MEDLINE In-Process/ePubs Ahead of Print, Embase, Cochrane Controlled Clinical Trial Register (Central), PubMed, and CINAHL was conducted, from database inception to May 29, 2019.

**Study Selection:**

Randomized clinical trials of interventions for adults with moderate to severe ARDS that used lung protective ventilation. No language restrictions were applied.

**Data Extraction and Synthesis:**

Data were independently extracted by 2 reviewers and synthesized with Bayesian random-effects network meta-analyses.

**Main Outcomes and Measures:**

The primary outcome was 28-day mortality. Barotrauma was a secondary outcome.

**Results:**

Among 25 randomized clinical trials evaluating 9 interventions, 2686 of 7743 patients (34.6%) died within 28 days. Compared with lung protective ventilation alone, prone positioning and venovenous extracorporeal membrane oxygenation were associated with significantly lower 28-day mortality (prone positioning: risk ratio, 0.69; 95% credible interval, 0.48-0.99; low quality of evidence; venovenous extracorporeal membrane oxygenation: risk ratio, 0.60; 95% credible interval, 0.38-0.93; moderate quality of evidence). These 2 interventions had the highest ranking probabilities, although they were not significantly different from each other. Among 18 trials reporting on barotrauma, 448 of 6258 patients (7.2%) experienced this secondary outcome. No intervention was superior to any other in reducing barotrauma, and each represented low to very low quality of evidence.

**Conclusions and Relevance:**

This network meta-analysis supports the use of prone positioning and venovenous extracorporeal membrane oxygenation in addition to lung protective ventilation in patients with ARDS. Moreover, venovenous extracorporeal membrane oxygenation may be considered as an early strategy for adults with severe ARDS receiving lung protective ventilation.

## Key Points

**Question:**

What is the relative association of management strategies for adult patients with moderate to severe acute respiratory distress syndrome with mortality and barotrauma?

**Findings:**

In this systematic review and network meta-analysis of 25 randomized clinical trials including 7743 patients, venovenous extracorporeal membrane oxygenation and prone positioning were associated with significantly lower 28-day mortality compared with lung protective ventilation alone. Moreover, venovenous extracorporeal membrane oxygenation was the highest-ranked intervention associated with a reduction in 28-day mortality among the 9 interventions evaluated.

**Meaning:**

These findings support the use of prone positioning in patients with moderate to severe acute respiratory distress syndrome and venovenous extracorporeal membrane oxygenation in patients with severe acute respiratory distress syndrome.

## Teaser

This systematic review and network meta-analysis compares and ranks different therapeutic strategies to identify the best intervention associated with a reduction in mortality in adult patients with moderate to severe acute respiratory distress syndrome (ARDS).

## Introduction

Acute respiratory distress syndrome (ARDS) is a lethal condition whereby the lung is injured by direct (eg, pneumonia or aspiration) or indirect (eg, extrapulmonary sepsis) insults. Clinically, patients with ARDS develop severe hypoxemia and/or hypercapnia, and most die of sepsis or multiorgan failure rather than from refractory respiratory failure. Acute respiratory distress is an important public health problem. A global epidemiologic study^1^ reported that 10.4% of total intensive care unit admissions and 23.4% of all patients who were intubated had ARDS, with an associated hospital mortality of 40%.

Since the first description of ARDS in 1967,^2^ a number of approaches to its management have been evaluated and used clinically.^3,4^ Despite more than 50 years of research, to our knowledge, none of the treatments currently available are aimed directly at the pathophysiological mechanism resulting in acute respiratory failure (ie, increased alveolar capillary permeability); current approaches are mainly supportive. Perhaps most important has been the recognition that although mechanical ventilation is critical for the survival of patients with ARDS, it can also be injurious (ie, ventilator-induced lung injury).^5^ Indeed, lung protective ventilation (LPV), using low tidal volumes and airway pressures to mitigate ventilator-induced lung injury, is considered the mainstay of management in patients with ARDS.^6^

In 2017, clinical practice guidelines on mechanical ventilation in adult patients with ARDS were published for 6 individual interventions, as follows: (1) LPV, (2) higher positive end-expiratory pressure (PEEP), (3) lung recruitment maneuvers (RMs), (4) high-frequency oscillatory ventilation (HFOV), (5) prone positioning, and (6) venovenous extracorporeal membrane oxygenation (VV ECMO).^6^ However, for clinicians, choosing between potentially efficacious treatments can be challenging if the treatments have not been directly compared in clinical trials (eg, prone positioning vs VV ECMO). Moreover, 4 randomized clinical trials (RCTs)—the Alveolar Recruitment for Acute Respiratory Distress Syndrome Trial (ART),^7^ the ECMO to Rescue Lung Injury in Severe ARDS (EOLIA) trial,^8^ the Esophageal Pressure-Guided Ventilation 2 (EPVent2) trial,^9^ and the Reevaluation of Systemic Early Neuromuscular Blockade (ROSE) trial^10^—have been published since the guidelines were completed. Therefore, we conducted a systematic review and network meta-analysis to compare different therapeutic strategies simultaneously to identify the best strategy associated with a reduction in mortality and to rank those therapeutic modalities for adult patients with moderate to severe ARDS.

## Methods

### Eligibility Criteria, Literature Search, and Study Selection

We followed the steps outlined by the Cochrane Collaboration^11^ and the Preferred Reporting Items for Systematic Reviews and Meta-analyses (PRISMA) reporting guideline.^12^ We included RCTs or quasi-RCTs enrolling adult patients (aged ≥18 years) with moderate to severe ARDS who received mechanical ventilation in the intensive care unit.^13^ For studies using the American-European Consensus Conference to diagnose ARDS, we checked Pao_2_-to-fraction of inspired oxygen (Fio_2_) ratio of inclusion criteria or the mean and distribution of Pao_2_/Fio_2_ ratio in each trial to determine the severity of ARDS among enrolled patients. We included interventions available for moderate to severe ARDS, either alone or in combination with LPV or another intervention. We defined LPV as a mechanical ventilation using low tidal volume of 4 to 8 mL/kg of predicted body weight. The interventions that we considered a priori were LPV, open lung strategies (ie, RM or PEEP), neuromuscular blockade (NMBA), inhaled nitric oxide (INO), HFOV, prone positioning, and VV ECMO. Participants in the comparator group could also have received 1 cointervention (eg, NMBA), as described above. Our primary outcome was 28-day mortality. If not reported explicitly, we identified or calculated 28-day mortality from Kaplan-Meier curves (using Digitaliser v10.9 [Engauge]) or from the closest reported time point, assuming constant mortality rate over time. Our prespecified secondary outcome was barotrauma at any time point. Inclusion and exclusion criteria are summarized in eAppendix 1 in the Supplement.

We performed an electronic search of MEDLINE, MEDLINE In-Process/ePub Ahead of Print, Embase, Cochrane Controlled Clinical Trial Register (Central) (via the Ovid search interface), PubMed (via the National Library of Medicine and excluding Medline records), and CINAHL (via EbscoHost) from database inception to May 29, 2019, using a sensitive search strategy (eAppendix 2 in the Supplement). We used controlled vocabulary terms (when available), text words, and keywords. No language restrictions were applied. We screened the reference lists of key articles for additional potentially relevant articles.

Two reviewers (H.A. and K.U.) independently identified and assessed potentially eligible studies for inclusion in the review, and any disagreement and discrepancies were resolved by discussion with and adjudication by a third reviewer (E.F.). Cohen κ was reported for agreement between the 2 reviewers.^14^ Data extraction and risk-of-bias assessment were also performed independently in duplicate by 2 authors (H.A. and K.U.).

### Data Extraction and Risk-of-Bias Assessment

A standardized, piloted data collection form designed for this systematic review was developed for data extraction. Risk of bias for each eligible study was determined using the Cochrane risk-of-bias tool.^11^ The certainty of evidence for the network meta-analysis was assessed and determined using the GRADE (Grading of Recommendations Assessment, Development, and Evaluation) tool for network meta-analysis.^15^ The risk of bias was graded as low, high, or unclear on the basis of each study’s randomization, allocation concealment, blinding of outcome assessment, losses to follow-up, treatment of withdrawals, and selective reporting. For performance and detection bias domains, we judged that, because mortality is objective, it was unlikely to be influenced by lack of blinding as long as a strict protocol for both groups was provided. To determine imprecision, a sample size required to detect a 30% relative risk reduction (optimal information size) was calculated for each comparison for each outcome based on a total event rate in the control group.^16,17,18^ Funnel plots were used to assess publication bias for each arm of the comparison, and further statistical analysis of funnel plot asymmetry was planned if there were more than 10 trials in each arm.^11^ Assessment of heterogeneity, consistency, and intransitivity are described in eAppendix 3 in the Supplement.

### Statistical Analysis

Standard pairwise meta-analysis methods with random-effect models were used to analyze interventions of eligible RCTs directly, where forest plots were constructed with subsequent calculation of risk ratios and 95% CIs for effect size. The *I*^2^ statistic was used to assess statistical heterogeneity in pairwise meta-analysis (eAppendix 3 in the Supplement). For network meta-analysis, we calculated effect sizes by determining risk ratios and 95% credible intervals (CrI) by the Bayesian hierarchical random-effects model, using Markov chain Monte Carlo simulation with noninformative prior distributions.^19,20,21,22^ For multiarm trials, correction of the treatment effects between arms was taken into account.^23^ Generalized linear models with a log-link function were applied for the analysis, with 4 chains and 2 000 000 iterated simulations, discarding the initial 1 500 000 iterations as burn-in. Potential scale reduction factor derived from the Brooks-Gelman-Rubin diagnostic was used for assessment of model convergence.^24^ Model fit was assessed by residual deviance, leverage, and the deviance information criterion.^25^ Consistency (ie, between-trial differences in the underlying treatment effects between comparisons) was assessed by the node-splitting method (ie, exploring differences between the treatment effects estimated by direct evidence and treatment effects estimated using indirect evidence), and transitivity (ie, the assumption that all treatments are equally likely candidates for the patients in the network) between comparisons was assessed by inspection of differences in potential effect modifiers (eAppendix 3 in the Supplement).

A rank statistic was determined and represented in a rankogram to illustrate the probability that a chosen treatment of all eligible interventions to be investigated was associated with the best, second best, and so on reduction in mortality.^26^ We also used the surface under cumulative ranking (SUCRA) curve to provide a numerical ranking of the association of all treatments with reduction in mortality, from 0 (certain to be the worst) to 100 (certain to be the best).^26^

Sensitivity analyses were conducted by excluding trials with high risk of bias and trials without a description of cointerventions. Model fit excluding small-sized trials was assessed as a sensitivity analysis. The other sensitivity analysis was to use Poisson models in the network meta-analysis for the primary outcome to adjust for different follow-up periods.^27^ Preplanned subgroup analysis was not conducted for the primary and secondary outcomes to assess the association of the distribution of outcome modifiers, including age and ARDS severity (ie, Pao_2_/Fio_2_ ratio), because network metaregression with treatment by covariate interactions showed none of the interventions included in our study were affected by age and ARDS severity at the study level (eAppendix 4 and eFigure 1 in the Supplement).

All statistical significance testing was 2-sided, and *P* < .05 was considered statistically significant. RevMan version 5.1 (The Nordic Cochrane Centre) was used to generate funnel plots and the risk-of-bias tables. Statistical analyses were conducted using R version 3.5.1 (The R Foundation) with R packages gemtc, coda, pcnetmeta, and rjags and using Just Another Giggs Samples version 4.3.0 (JAGS).

## Results

**Figure 1.**
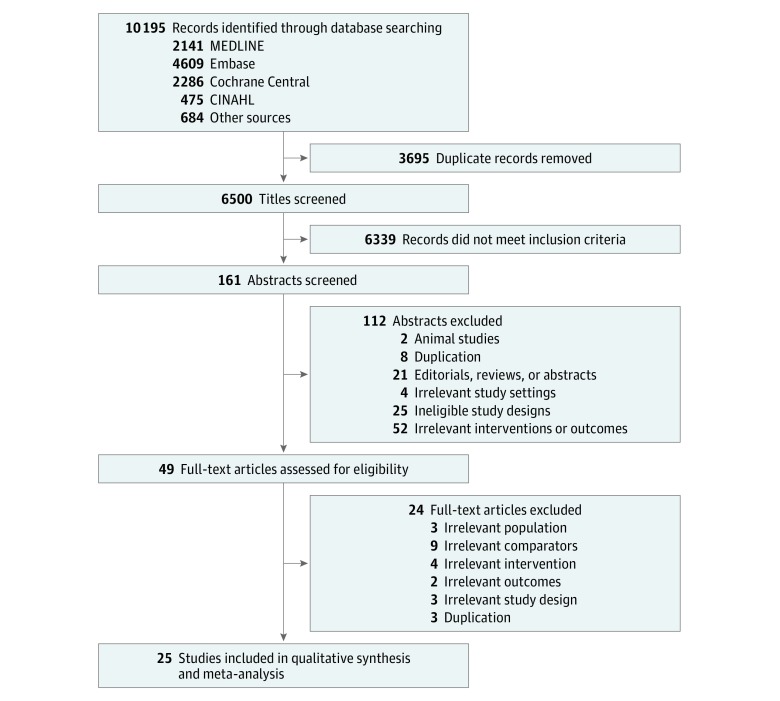
PRISMA Flow Diagram

**Figure 2.**
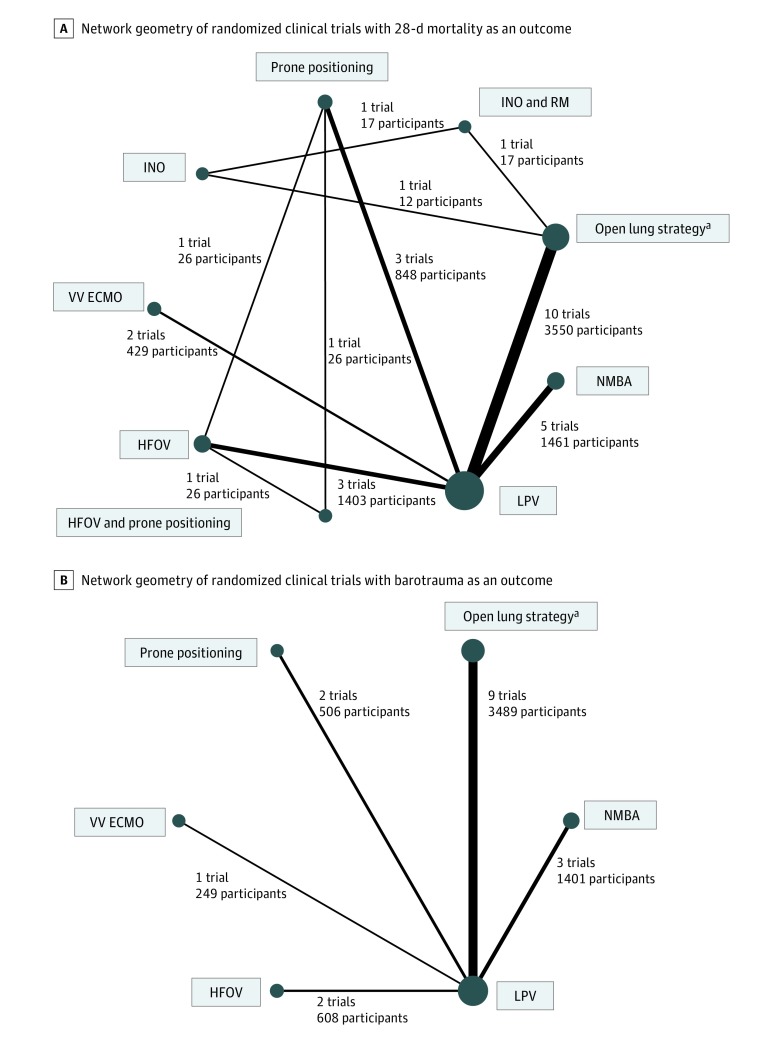
Network Geometry and Ranking Probabilities for the Association of Interventions With Outcomes Network geometry shows the interventions as nodes and the direct comparisons as connections between the nodes. Larger node size indicates a larger total number of participants in arms including that intervention, and thicker connection indicates a larger number of trials investigating that comparison. HFOV indicates high-frequency oscillatory ventilation; INO, inhaled nitric oxide; LPV, lung protective ventilation; NMBA, neuromuscular blockade; RM, recruitment maneuver; and VV ECMO, venovenous extracorporeal membrane oxygenation. ^a^Open lung strategy using RM and/or higher positive end-expiratory pressure.

We identified 10 195 records in our electronic search (Figure 1). After screening by title and abstract, we obtained full-text articles for 49 citations that were potentially eligible for inclusion. We included 25 studies (7753 participants; range, 20-1010 participants) in this review (Table 1),^7,8,9,10,28,29,30,31,32,33,34,35,36,37,38,39,40,41,42,43,44,45,46,47,48^ while 24 studies did not meet our inclusion criteria (eTable 1 in the Supplement). There was near-perfect agreement on study inclusion between the 2 reviewers (κ = 0.97). Overall, 9 interventions were investigated (Figure 2) (eFigure 2 and eFigure 3 in the Supplement): LPV (23 trials),^7,8,9,10,28,29,30,31,32,33,34,35,36,37,38,39,40,41,42,43,44,45,46^ an open lung strategy using RM and/or higher PEEP (10 trials),^7,9,39,40,41,42,43,44,45,46^ NMBA using a 48-hour infusion of cisatracurium (5 trials),^10,35,36,37,38^ INO (1 trial),^47^ INO with RM (1 trial),^47^ HFOV (3 trials),^28,29,30^ HFOV with prone positioning (1 trial),^48^ prone positioning (3 trials),^32,33,34^ and VV ECMO (2 trials).^8,31^

**Table 1.** Summary of Identified 25 Studies

Source	Study Period	Study Sites (Country)	No. of Patients	Inclusion Criteria of Pao_2_/Fio_2_ in AECC Definition or ARDS Severity in Berlin Definition	Protocolized Vt Setting	Experimental/Control Age, Mean (SD), y	Experimental/Control Pao_2_/Fio_2 _Ratio at Enrollment, Mean (SD)	Primary Outcome	Experimental/Control, 28-d Mortality, %	Overall Risk of Bias
**HFOV**										
Ferguson et al,^28^ 2013	2007-2012	39 Centers (Canada, the United States, Saudi Arabia, Chile, and India)	548	Pao_2_/Fio_2_ ≤ 200	6 mL/kg PBW	55 (16)/54 (16)	121 (46)/114 (38)	Hospital mortality	40.4/28.6	Unclear
Young et al,^29^ 2013	2007-2012	29 Centers (United Kingdom)	795	Pao_2_/Fio_2_ ≤ 200	6-8 mL/kg PBW	55 (19)/56 (16)	113 (37)/113 (38)	30-d Mortality	41.7/41.1	Low
Mohamed and Mohamed,^30^ 2016	2007-2011	Not specified (Saudi Arabia)	60	Pao_2_/Fio_2_ ≤ 200	4-6 mL/kg PBW	46 (10)/44 (12)	Not reported	30-d Mortality	53.3/56.7	High
**VV ECMO**										
Peek et al,^31^ 2009	2001-2006	68 Hospitals (United Kingdom)	180	Severe^a^	4-8 mL/kg PBW	40 (13)/40 (13)	76 (30)/75 (36)	6-mo Mortality	22.2/46.7	Unclear
Combes et al,^8^ 2018	2011-2017	23 Centers (France)	249	Pao_2_/Fio_2_ < 200	6 mL/kg of PBW	52 (14)/54 (13)	73 (30)/72 (24)	60-d Mortality	25.8/36.8	Unclear
**Prone Positioning**										
Fernandez et al,^32^ 2008	2003-2004	17 Hospitals (Spain)	40	Pao_2_/Fio_2_ ≤ 200	6-8 mL/kg PBW	54 (18)/55 (15)	153 (59)/158 (84)	60-d Mortality	19.0/26.3	Unclear
Taccone et al,^33^ 2009	2004-2008	25 Centers (Italy and Spain)	342	Pao_2_/Fio_2_ ≤ 200	≤8 mL/kg PBW	Not available	Not available	28-d Mortality	31.0/32.8	Unclear
Guérin et al,^34^ 2013	2008-2011	27 Centers (France and Spain)	466	Pao_2_/Fio_2_ < 150	6 mL/kg PBW	58 (16)/60 (16)	100 (30)/100 (20)	28-d Mortality	16.0/32.8	Low
**NMBA**										
Gainnier et al,^35^ 2004	2000-2001	4 Centers (France)	56	Pao_2_/Fio_2_ < 150	6-8 mL/kg PBW	60 (18)/62 (15)	130 (34)/119 (31)	Pao_2_/FIO_2_ during 120 h after randomization	35.7/60.7	Low
Forel et al,^36^ 2006	2002-2003	3 Centers (France)	36	Pao_2_/Fio_2_ ≤ 200	4-8 mL/kg PBW	52 (16)/61 (18)	Not available	Pulmonary and systemic inflammatory response	27.8/55.6	Low
Papazian et al,^37^ 2010	2006-2008	20 Centers (France)	339	Pao_2_/Fio_2_ < 150	6-8 mL/kg PBW	58 (16)/58 (15)	106 (36)/115 (41)	90-d Mortality	23.7/33.3	Low
Guervilly et al,^38^ 2017	2012-2014	2 Centers (France)	24	Moderate	6 mL/kg PBW	72 (63-79)/60 (52-75)^b^	158 (131-185)/150 (121-187)^b^	Change in transpulmonary pressure	38.5/27.3	Low
Moss et al,^10^ 2019	2016-2018	48 Centers (United States)	1006	Pao_2_/Fio_2_ < 150	6 mL/kg PBW	56.6 (14.7)/55.1 (15.9)	98.7 (27.9)/99.5 (27.9)	90-d Mortality	36.7/37.0	Low
**Open Lung Strategy Using RM and/or Higher PEEP**										
Brower et al,^39^ 2004	1999-2002	23 Centers (United States)	549	All^c^	6 mL/kg PBW	54 (17)/49 (17)	151 (67)/165 (77)	Hospital mortality	24.2/28.7^c^	High
Meade et al,^40^ 2008	2000-2006	30 Centers (Canada, Australia, and Saudi Arabia)	983	Pao_2_/Fio_2_ ≤ 250^c^	6 mL/kg PBW with allowances for 4-8 mL	55 (17)/57 (17)	145 (48)/145 (49)	Hospital mortality		Low
Mercat et al,^41^ 2008	2002-2005	37 Centers (France)	767	All^c^	6 mL/kg PBW	60 (16)/60 (15)	144 (58)/143 (57)	28-d Mortality		Low
Talmor et al,^42^ 2008	2004-2007	1 Center (United States)	61	All^d^	6 mL/kg PBW	55 (16)/51 (23)	147 (56)/145 (57)	Pao_2_/FIO_2_ 72 h after randomization	16.7/38.7	Low
Huh et al,^43^ 2009	2004-2006	1 Center (Korea)	57	Pao_2_/Fio_2_ ≤ 200	6 mL/kg PBW with allowances up to 8 mL	55 (4)/62 (2)	115 (9)/111 (6)	Pao_2_/FIO_2_	40.0/33.3	Unclear
Xi et al,^44^ 2010	2003-2006	14 Centers (China)	110	Pao_2_/Fio_2_ ≤ 200	6-8 mL/kg PBW	62 (16)/66 (15)	94 (69-150)/120 (88-140)^b^	ICU mortality	29.1/43.6	High
Hodgson et al,^45^ 2011	2008-2009	1 Center (Australia)	20	Pao_2_/Fio_2_ ≤ 200	≤6 mL/kg PBW	60 (5)/58 (4)	155 (8)/149 (12)	IL-6 level	50.0/20.0	Low
Kacmarek et al,^46^ 2016	2007-2013	20 Centers (Spain, Brazil, South Korea, Peru, Chile, and United States)	200	Pao_2_/Fio_2_ ≤ 200	4-8 mL/kg PBW	52 (15)/53 (15)	121 (37)/114 (33)	60-d Mortality	22.2/26.7	Low
Cavalcanti et al,^7^ 2017	2011-2017	120 Centers (Brazil, Argentina, Colombia, Italy, Poland, Portugal, Malaysia, Spain, and Uruguay)	1010	Moderate to severe	4-6 mL/kg PBW	51 (17)/51 (17)	120 (44)/117 (42)	28-d Mortality	55.3/49.3	Low
Beitler et al,^9^ 2019	2012-2017	14 Centers (United States and Canada)	200	Moderate to severe	4-8 mL/kg PBW	58 (47-66)/57.5 (43-69)^b^	95 (73-129)/90 (69-123)^b^	Ranked composite incorporating death and days free of mechanical ventilation among survivors	30.6/32.4	Low
**INO and RM**										
Park et al,^47^ 2003	Not stated	1 Center (Korea)	23	Pao_2_/Fio_2_ ≤ 200	6 mL/kg PBW	52 (17)/50 (20)/59 (13)^e^	140 (27)/162 (19)/105 (14)^e^	Not specified	36.4/33.3/66.7^e^	High
**HFOV and Prone Positioning**										
Papazian et al,^48^ 2005	Not stated	1 Center (France)	39	Pao_2_/Fio_2_ < 150	6 mL/kg PBW	55 (15)/51 (12)/51 (9)^e^	106 (31)/101 (22)/103 (41)^e^	Not specified	30.8/23.1/38.5^e^	Unclear

Abbreviations: AECC, American-European Consensus Conference; ARDS, acute respiratory distress syndrome; HFOV, high-frequency oscillatory ventilation; Fio_2, _fraction of inspired oxygen; ICU, intensive care unit; IL-6, interleukin 6; INO, inhaled nitric oxide; NMBA, neuromuscular blockade; PBW, predicted body weight; PEEP, positive end-expiratory pressure; RM, recruitment maneuver; Vt, tidal volume; VV ECMO, venovenous extracorporeal membrane oxygenation.

^a^ Severe but potentially reversible respiratory failure, Murray score 3.0 or higher, or uncompensated hypercapnia with pH less than 7.20.

^b^ Median (interquartile range) reported.

^c^ A subsequent systematic review of individual patient-level data, including Brower et al,^39^ Meade et al,^40^ Mercat et al,^41^ reported the total number of events and patients who were diagnosed as having ARDS with Pao_2_/Fio_2_ less than 200. These data were extracted and used for further analyses in this study.

^d^ Nearly all enrolled patients had moderate or severe ARDS.

^e^ Study included 3 arms.

There was high risk of bias for 1 or more key domains in 4 studies (eFigures 4-6 in the Supplement).^30,39,44,47^ All trials specified standardized protocols for the implementation of each intervention and ventilatory management; thus, blinding domains were judged low for our primary outcome. As a result, 14 of 25 studies (56%) had a low risk of bias across all domains.^7,9,10,29,34,35,36,37,38,40,41,42,45,46^

Among the 25 studies, overall 28-day mortality was 34.6% (2686 of 7753 patients). Overall, LPV was the most frequently investigated intervention, whereas 3 interventions were investigated by only 1 trial (Figure 2). Compared with LPV alone, prone positioning (risk ratio, 0.69; 95% CrI, 0.48-0.98; low quality of evidence) and VV ECMO (risk ratio, 0.60; 95% CrI, 0.38-0.93; moderate quality of evidence) were associated with significantly lower 28-day mortality (Table 2) (eTable 2, eFigure 2, and eFigure 7 in the Supplement). Prone positioning prevented 124 more deaths per 1000 patients compared with LPV alone, and VV ECMO prevented 161 more deaths per 1000 patients compared with LPV alone (Table 2). The results of comparisons between all possible pairs of interventions with quality of evidence are summarized in eTable 3 in the Supplement. In a sensitivity analysis restricted to studies without a high risk of bias (12 studies; 4213 patients) and studies with a description of cointerventions (17 studies; 7196 patients), prone positioning and VV ECMO remained significantly associated with lower 28-day mortality (eTable 4 and eTable 5 in the Supplement). A sensitivity analysis using Poisson models showed that VV ECMO and prone positioning were significantly associated with reduced mortality (eTable 6 in the Supplement). Exclusion of small-sized trials did not change model fit in the network meta-analysis (eTables 7-9 in the Supplement).

**Table 2.** Summary of Findings for 28-Day Mortality

Comparison	No.		Network Risk Ratio (95% CrI)	Anticipated Absolute Effect^a^		Quality of Evidence
	Patients	Trials		With Intervention per 1000	Difference (95% CrI)	
LPV	NA	NA	1 [Reference]	401	NA	NA
VV ECMO	429	2	0.60 (0.38 to 0.93)	240	−161 (−249 to −28)	Moderate
HFOV	1403	3	1.12 (0.83 to 1.54)	449	48 (−68 to 200)	Low
HFOV and prone positioning	NA	Indirect evidence	0.53 (0.12 to 1.60)	212	−188 (−353 to 241)	Very low
NMBA	956	5	0.79 (0.57 to 1.02)	318	−83 (−173 to 8)	High
Open lung strategy^b^	3452	10	0.96 (0.77 to 1.14)	385	−16 (−92 to 56)	Low
INO and RM	NA	Indirect evidence	0.86 (0.22 to 3.83)	345	−56 (−313 to 599)	Very low
Prone positioning	848	3	0.69 (0.48 to 0.98)	277	−124 (−208 to −8)	Low
INO	NA	Indirect evidence	1.48 (0.42 to 6.08)	593	192 (−233 to 599)	Very low
VV ECMO	NA	NA	1 [Reference]	240	NA	NA
HFOV	NA	Indirect evidence	1.88 (1.12 to 3.24)	451	211 (29 to 538)	Low
HFOV and prone positioning	NA	Indirect evidence	0.87 (0.19 to 2.90)	209	−31 (−194 to 456)	Very low
NMBA	NA	Indirect evidence	1.30 (0.77 to 2.14)	312	72 (−55 to 274)	Very low
Open lung strategy^b^	NA	Indirect evidence	1.59 (0.99 to 2.56)	382	142 (−2 to 374)	Very low
INO and RM	NA	Indirect evidence	1.43 (0.34 to 6.60)	343	103 (−158 to 640)	Very low
Prone positioning	NA	Indirect evidence	1.15 (0.66 to 2.01)	276	36 (−82 to 242)	Very low
INO	NA	Indirect evidence	2.48 (0.67 to 10.90)	595	355 (−79 to 760)^b^	Very low
HFOV	NA	NA	1 [Reference]	435	NA	NA
HFOV and prone positioning	26	1	0.47 (0.11 to 1.44)	204	−230 (−387 to 191)	Low
NMBA	NA	Indirect evidence	0.70 (0.48 to 1.02)	304	−130 (−226 to 9)	Moderate
Open lung strategy^b^	NA	Indirect evidence	0.85 (0.58 to 1.19)	370	−65 (−183 to 83)	Low
INO and RM	NA	Indirect evidence	0.76 (0.18 to 3.50)	331	−104 (−357 to 565)	Very low
Prone positioning	26	1	0.61 (0.39 to 0.95)	265	−170 (−265 to −22)	Moderate
INO	NA	Indirect evidence	1.32 (0.35 to 5.51)	574	139 (−283 to 565)	Very low
HFOV and prone positioning	NA	NA	1 [Reference]	231	NA	NA
NMBA	NA	Indirect evidence	1.48 (0.45 to 6.63)	342	111 (−127 to 769)	Very low
Open lung strategy^b^	NA	Indirect evidence	1.80 (0.56 to 8.01)	416	184 (−102 to 769)	Very low
INO and RM	NA	Indirect evidence	1.67 (0.26 to 13.36)	386	155 (−171 to 769)	Very low
Prone positioning	26	1	1.31 (0.41 to 5.83)	303	72 (−136 to 769)	Low
INO	NA	Indirect evidence	2.93 (0.49 to 21.97)	677	446 (−118 to 769)	Very low
NMBA	NA	NA	1 [Reference]	314	NA	NA
Open lung strategy^b^	NA	Indirect evidence	1.22 (0.90 to 1.72)	383	69 (−31 to 226)	Very low
INO and RM	NA	Indirect evidence	1.10 (0.28 to 5.04)	345	31 (−226 to 686)	Very low
Prone positioning	NA	Indirect evidence	0.88 (0.56 to 1.40)	276	38 (−138 to 126)	Very low
INO	NA	Indirect evidence	1.91 (0.51 to 8.23)	600	286 (−154 to 686)	Very low
Open lung strategy^b^	NA	NA	1 [Reference]	403	NA	NA
INO and RM	17	1	0.90 (0.24 to 3.91)	363	−40 (−306 to 597)	Very low
Prone positioning	NA	Indirect evidence	0.72 (0.48 to 1.11)	290	−113 (−210 to 34)	Very low
INO	12	1	1.55 (0.44 to 6.28)	625	222 (−226 to 597)	Very low
INO and RM	NA	NA	1 [Reference]	364	NA	NA
Prone positioning	NA	Indirect evidence	0.80 (0.18 to 3.22)	291	−73 (−299 to 636)	Very low
INO	17	1	1.74 (0.51 to 5.94)	633	269 (−128 to 636)	Very low
Prone positioning	NA	NA	1 [Reference]	242	NA	NA
INO	NA	Indirect evidence	2.15 (0.59 to 8.99)	520	278 (−99 to 758)	Very low

Abbreviations: CrI, credible interval; HFOV, high-frequency oscillatory ventilation; INO, inhaled nitric oxide; LPV, lung protective ventilation; NA, not applicable; NMBA, neuromuscular blockade; RM, recruitment maneuver; VV ECMO, venovenous extracorporeal membrane oxygenation.

^a^ To compute anticipated absolute effect, risk ratio is less than or equal to 1 divided by event rate in the reference group (ie, 1/average control risk).

^b^ Open lung strategy using RM and/or higher positive end-expiratory pressure.

The incidence of barotrauma was 7.2% (448 of 6253 patients) from 17 trials evaluating 6 interventions (Figure 2). There were no significant differences between interventions in the risk of barotrauma, with variable quality of evidence (Table 3) (eTable 10, eTable 11, eFigure 3, and eFigure 7 in the Supplement).

**Table 3.** Summary of Findings for Barotrauma

Comparison	No.		Network Risk Ratio (95% CrI)	Anticipated Absolute Effect^a^		Quality of Evidence
	Patients	Trials		With Intervention per 1000	Difference (95% CrI)	
LPV	NA	NA	1 [Reference]	68	NA	NA
VV ECMO	249	1	1.19 (0.24 to 5.88)	81	13 (−52 to 332)	Moderate
HFOV	608	2	1.69 (0.55 to 7.14)	115	47 (−31 to 417)	Low
NMBA	896	3	0.47 (0.18 to 1.03)	32	−36 (−56 to 2)	Low
Open lung strategy^b^	3391	9	1.11 (0.54 to 1.84)	75	7 (−31 to 57)	Low
Prone positioning	506	2	0.78 (0.19 to 2.32)	53	−15 (−55 to 90)	Low
VV ECMO	NA	NA	1 [Reference]	145	NA	NA
HFOV	NA	Indirect evidence	1.35 (0.21 to 8.47)	196	51 (−115 to 855)	Very low
NMBA	NA	Indirect evidence	0.39 (0.07 to 1.86)	56	−89 (−135 to 125)	Very low
Open lung strategy^b^	NA	Indirect evidence	0.94 (0.18 to 3.67)	136	−7 (−119 to 387)	Very low
Prone positioning	NA	Indirect evidence	0.65 (0.08 to 3.55)	94	−51 (−133 to 370)	Very low
HFOV	NA	NA	1 [Reference]	134	NA	NA
NMBA	NA	Indirect evidence	0.29 (0.05 to 0.92)	39	−95 (−128 to −11)	Very low
Open lung strategy^b^	NA	Indirect evidence	0.69 (0.14 to 1.88)	92	−42 (−115 to 118)	Very low
Prone positioning	NA	Indirect evidence	0.48 (0.06 to 1.89)	64	−70 (−126 to 119)	Very low
NMBA	NA	NA	1 [Reference]	25	NA	NA
Open lung strategy^b^	NA	Indirect evidence	2.36 (0.79 to 6.91)	59	34 (−5 to 148)	Very low
Prone positioning	NA	Indirect evidence	1.66 (0.34 to 7.03)	41	16 (−17 to 151)	Very low
Open lung strategy^b^	NA	NA	1 [Reference]	59	NA	NA
Prone positioning	NA	Indirect evidence	0.70 (0.17 to 2.61)	41	−18 (−49 to 95)	Very low

Abbreviations: CrI, credible interval; HFOV, high-frequency oscillatory ventilation; LPV, lung protective ventilation; NA, not applicable; NMBA, neuromuscular blockade; VV ECMO, venovenous extracorporeal membrane oxygenation.

^a^ To compute anticipated absolute effect, risk ratio is less than or equal to 1 divided by event rate in the reference group (ie, 1/average control risk).

^b^ Open lung strategy using recruitment maneuver and/or higher positive end-expiratory pressure.

For mortality, both VV ECMO and prone positioning were ranked highly on the basis of SUCRA, with VV ECMO being highest (0.82). However, the ranking probability for VV ECMO did not differ significantly from prone positioning (eFigure 8 in the Supplement). For barotrauma, NMBA had the highest SUCRA (0.93), although no intervention was significantly different from any other in reducing barotrauma in studies reporting this outcome (eFigure 8 in the Supplement).

There were 36 direct or indirect comparisons for the primary outcome among 25 studies (eTable 3 in the Supplement). Only 7 comparisons were not affected by moderate or high heterogeneity (eTable 3 in the Supplement). Comparisons of LPV, HFOV, and open lung strategy using RM and/or higher PEEP in an arm with any intervention in the other arm met the optimal information size for imprecision. Node splitting found no significant inconsistency in 3 comparisons (HFOV vs LPV, prone positioning vs LPV, and prone positioning vs HFOV) (eTable 12 in the Supplement). Intransitivity was not found based on network metaregression with treatment by covariate interactions to measure potential effect modifiers. Based on these findings in the primary outcome among all 36 comparisons, the quality of evidence for network effects estimates was judged as high in 1 comparison, moderate in 4, low in 6, and very low in 25 (eTable 3 in the Supplement).

No comparison for barotrauma had a high risk of bias or met the optimal information size in imprecision (eTable 11 in the Supplement). All comparisons, except VV ECMO vs LPV, showed moderate to high heterogeneity. Because there was no closed loop of interventions for barotrauma, the assumption of consistency in any comparison cannot be violated. Intransitivity was not found. The quality of evidence for network effects estimates in barotrauma was judged as moderate in 1 comparison, low in 4, and very low in 10 (eTable 11 in the Supplement).

## Discussion

Our systematic review and network meta-analysis of 25 RCTs, which included 7753 patients and 9 interventions, found that prone positioning was associated with significantly lower 28-day mortality in patients with moderate ARDS compared with LPV alone. In patients with severe ARDS, VV ECMO was associated with significantly lower 28-day mortality. Furthermore, these interventions were highly ranked, with VV ECMO being the highest, although there were no significant differences in ranking probabilities between these interventions. No intervention was superior to any other in reducing barotrauma.

While the use of LPV remains the mainstay of supportive care in patients with ARDS,^6^ LPV alone may be insufficient to maintain adequate gas exchange or prevent ventilator-induced lung injury, and adjunctive interventions may be required. While many of these adjunctive interventions have been evaluated in RCTs or meta-analyses, to our knowledge, there are limited data comparing their relative efficacy with each other. Wang et al^49^ assessed 26 ventilatory strategies in their network meta-analysis for ARDS but divided the interventions into complex groupings, making clinical interpretation difficult. Moreover, they made comparisons with a strategy of higher tidal volumes that has little clinical relevance in the current management of patients with ARDS.^49^ In contrast, we decided to focus on a limited number of interventions that are commonly used in patients with moderate to severe ARDS^1^ rather than include all possibilities to provide the most relevant guidance for clinicians at the bedside.

Our results are consistent with the strong recommendations from the American Thoracic Society, European Society of Intensive Care Medicine, and Society of Critical Care Medicine clinical practice guidelines^6^ based on conventional (pairwise) meta-analyses. Specifically, our study supports the use of prone positioning (ie, significant reduction in mortality and high ranking) as well as the strong recommendation against the routine use of HFOV (ie, increased mortality and low ranking). The conditional recommendations for RMs or higher PEEP did not include the results of the ART^7^ or the EPVent2 trial.^9^ Our pooled results did not find an association of RMs or higher PEEP with mortality. Importantly, the guideline could not make a recommendation on the use of VV ECMO in patients with severe ARDS owing to insufficient data. Our study supports the earlier consideration of VV ECMO along with LPV in patients with severe ARDS, consistent with the results of the EOLIA trial,^8^ the post hoc Bayesian reanalysis of that trial,^50^ and the updated meta-analysis of VV ECMO studies.^51^ The results for INO, which were not included in the guideline, are consistent with the 2016 meta-analysis^52^ that showed an increased risk for renal failure with no significant mortality benefit for INO. Finally, although the most recent meta-analysis of NMBA, to our knowledge, showed a reduction in mortality,^53^ our study results are consistent with the results of the ROSE trial,^10^ in which NMBA did not improve mortality of patients with moderate to severe ARDS.

By including the ART,^7^ EPVent2,^9^ EOLIA,^8^ and ROSE trials^10^ in our network meta-analysis, our findings could inform future updates of the clinical practice guidelines and decision-making process at the bedside. Given the limited clinical interpretation of SUCRA (relative efficacy assessment on eligible interventions),^26^ our findings demonstrate that prone positioning and VV ECMO should be considered early, with implementation of these interventions influenced by individual patient characteristics or available clinical resources. Our preplanned metaregression confirmed that ARDS severity (ie, aggregated Pao_2_/Fio_2_ ratio at study level) did not affect the findings of these analyses. However, VV ECMO should be restricted to severe ARDS, since the 2 included RCTs of VV ECMO only recruited patients with severe ARDS. Moreover, in addition to the statistical heterogeneity in severity among eligible studies, considerations such as the invasiveness, availability of resources, and need for clinical experience may also limit the use of VV ECMO to the patients with the most severe ARDS in high-volume centers.^54^ Therefore, prone positioning should be considered as a first-line approach for patients with moderate to severe ARDS in addition to LPV. Timely transfer to an ECMO-capable center and VV ECMO should be considered for patients with severe ARDS who have contraindications or who fail prone positioning, similar to the EOLIA trial.^8^ The strategy of HFOV combined with prone positioning was associated with a nonsignificant reduction in mortality but with a high SUCRA; however, inferences about its clinical efficacy and relevance remain uncertain owing to limitations from a single study with very small sample size.^48^ The lower rankings of the other 5 interventions (NMBA, HFOV, INO, INO with RM, and open lung strategy using RM and/or higher PEEP) suggests against their routine use.

### Limitations

This study has several limitations. First, the network meta-analysis depends on the assumption that population and intervention characteristics were largely similar across the included studies (ie, transitivity). Thus, an analysis including trials applying different thresholds of Pao_2_/Fio_2_ ratio (ie, severity of ARDS) for the patient recruitment is subject to bias. For example, the EOLIA trial only enrolled patients with severe ARDS. Furthermore, this assumption may also be violated by the difference in the period of those trials performed. We confirmed that there was no effect modification of Pao_2_/Fio_2_ ratio on treatment by covariate interaction by network metaregressions with Pao_2_/Fio_2_ ratio (eFigure 1 in the Supplement). However, the most robust method for evaluating the potential for effect modification by Pao_2_/Fio_2_ ratio would be with a meta-analysis of individual patient data. Second, some studies allowed or recommended cointerventions in some settings for both experimental and control groups (eg, prone positioning for certain Pao_2_/Fio_2_ ratios in a study of NMBA) but not in all participants. However, many studies did not explicitly describe the use of cointerventions in the protocol or results of their published articles. We conducted a sensitivity analysis excluding studies without a description of cointerventions, which was robust for the main analysis (eTable 5 and eTable 13 in the Supplement). Moreover, we attempted to address this limitation only by downrating the quality in GRADE assessment. Future trials are strongly encouraged to provide a detailed description of cointerventions used or to protocolize their implementation within the trial. Third, we only evaluated short-term mortality. However, we believe that this approach is theoretically justified because most interventions studied have the main rationale of reducing ventilator-induced lung injury and more immediate death. Future studies should include an evaluation of long-term mortality and functional outcomes. Fourth, the adverse effects of treatments were limited because of the nature of different interventions. We only assessed barotrauma, and there were differences in definition of barotrauma in each study. Fifth, although intervention from smaller studies (eg, INO, HFOV with prone positioning) can lead to imprecision of estimated effects (ie, wider CrIs), we confirmed that excluding such small-sized studies did not change overall model fitting of the network meta-analysis and the other main findings (eTable 7-9 in the Supplement). Sixth, we could not specify 28-day mortality in 5 of 25 trials (20%) and computed mortality assuming constant risk of death during the study period. An alternative way to address this issue was to use Poisson models in the network meta-analysis and use follow-up period as offset in the model. Using this approach, we found no differences between Poisson models and our primary analyses (eTable 6 in the Supplement). Seventh, we did not account for covariates (ie, age and Pao_2_/Fio_2_ ratio) in the final models of network meta-analysis because we did not find treatment by covariate interaction from network metaregressions with these covariates. However, these findings were based on study-level data instead of individual patient data, so aggregation bias still remains possible. Eighth, a meta-analysis with individual patient data may be desirable to further separate the effectiveness of each intervention and explore outcomes in important patient subgroups. Despite these limitations, the use of network meta-analysis enabled us to compare clinically relevant management strategies in patients with ARDS. Ninth, we used robust methods as recommended by the Cochrane Collaboration, PRISMA, and GRADE for network meta-analysis.^11,12,15^

## Conclusions

This network meta-analysis supports the use of prone positioning and VV ECMO in addition to LPV in patients with moderate to severe ARDS and severe ARDS, respectively. In addition, our results are consistent with recent data suggesting that VV ECMO may be considered as an early strategy for adults with severe ARDS.

## Notes

Supplement. **eAppendix 1.** Summary of Inclusion and Exclusion Criteria **eAppendix 2.** Updated Search Strategy in MEDLINE, Embase, Cochrane, PubMed, and CINAHL **eAppendix 3.** Assessment of Heterogeneity, Consistency, and Intransitivity **eAppendix 4.** Network Metaregression With Treatment by Covariate Interactions to Measure the Impact of Age and ARDS Severity **eFigure 1.** Results of Network Metaregression for Age (a) and ARDS Severity (b) **eFigure 2.** Forest Plots of Direct, Indirect, and Pooled Comparisons for Primary Outcome **eFigure 3.** Forest Plots of Direct, Indirect, and Pooled Comparisons for Secondary Outcome **eFigure 4.** Risk-of-Bias Graph for All Eligible Studies **eFigure 5.** Risk-of-Bias Summary for All Eligible Studies **eFigure 6.** Funnel Plots for Primary (a) and (b) Secondary Outcomes **eFigure 7.** Gelman Plots for Model Convergence With 4 Chains and 2 000 000 Iterated Simulations, Discarding the Initial 1 500 000 Iterations as Burn-in on Primary (a) and (b) Secondary Outcomes **eFigure 8.** Ranking Probabilities for the Association of Interventions With Outcomes **eTable 1. **Detailed Explanation for Excluded Studies With Full-Text Assessment **eTable 2.** Comparison of the Included Interventions in Risk Ratio (95% CrI) for 28-Day Mortality **eTable 3.** Network Meta-analysis for 28-Day Mortality and Quality of Evidence Assessment **eTable 4.** Results of Sensitivity Analysis for 28-Day Mortality **eTable 5.** Results of Preliminary Analysis Excluding Studies Without Description of Cointerventions **eTable 6.** Results of Analysis Using Poisson Models for the Primary Outcome Adjusting for Different Timing of the Measurement **eTable 7.** Potential Scale Reduction Factor From Brooks-Gelman-Rubin Diagnostic for Model Convergence on Primary (a) and (b) Secondary Outcomes **eTable 8.** Assessment of Model Fits for Primary (a) and (b) Secondary Outcomes **eTable 9.** Results of Assessment on Small Sample Size Effects **eTable 10.** Comparison of the Included Interventions in Risk Ratio (95% CrI) for Barotrauma **eTable 11.** Network Meta-analysis for Barotrauma and Quality of Evidence Assessment **eTable 12.** Results of Node-Splitting Models **eTable 13.** Summary of Cointerventions in Included Studies **eReferences**
