# Supplementary material for: Assessment of Therapeutic Interventions and Lung Protective Ventilation in Patients With Moderate to Severe Acute Respiratory Distress Syndrome: A Systematic Review and Network Meta-analysis
Source: JAMA Netw Open. 2019 Jul 31;2(7):e198116. doi: 10.1001/jamanetworkopen.2019.8116 (PMC6669780; doi:10.1001/jamanetworkopen.2019.8116)
Supplement: Supplement. — eAppendix 1. Summary of Inclusion and Exclusion Criteria eAppendix 2. Updated Search Strategy in MEDLINE, Embase, Cochrane, PubMed, and CINAHL eAppendix 3. Assessment of Heterogeneity, Consistency, and Intransitivity eAppendix 4. Network Metaregression With Treatment by Covariate Interactions to Measure the Impact of Age and ARDS Severity eFigure 1. Results of Network Metaregression for Age (a) and ARDS Severity (b) eFigure 2. Forest Plots of Direct, Indirect, and Pooled Comparisons for Primary Outcome eFigure 3. Forest Plots of Direct, Indirect, and Pooled Comparisons for Secondary Outcome eFigure 4. Risk-of-Bias Graph for All Eligible Studies eFigure 5. Risk-of-Bias Summary for All Eligible Studies eFigure 6. Funnel Plots for Primary (a) and (b) Secondary Outcomes eFigure 7. Gelman Plots for Model Convergence With 4 Chains and 2 000 000 Iterated Simulations, Discarding the Initial 1 500 000 Iterations as Burn-in on Primary (a) and (b) Secondary Outcomes eFigure 8. Ranking Probabilities for the Association of Interventions With Outcomes eTable 1. Detailed Explanation for Excluded Studies With Full-Text Assessment eTable 2. Comparison of the Included Interventions in Risk Ratio (95% CrI) for 28-Day Mortality eTable 3. Network Meta-analysis for 28-Day Mortality and Quality of Evidence Assessment eTable 4. Results of Sensitivity Analysis for 28-Day Mortality eTable 5. Results of Preliminary Analysis Excluding Studies Without Description of Cointerventions eTable 6. Results of Analysis Using Poisson Models for the Primary Outcome Adjusting for Different Timing of the Measurement eTable 7. Potential Scale Reduction Factor From Brooks-Gelman-Rubin Diagnostic for Model Convergence on Primary (a) and (b) Secondary Outcomes eTable 8. Assessment of Model Fits for Primary (a) and (b) Secondary Outcomes eTable 9. Results of Assessment on Small Sample Size Effects eTable 10. Comparison of the Included Interventions in Risk Ratio (95% CrI) for Barotrauma eTable 11. Network Meta- [file jamanetwopen-2-e198116-s001.pdf]

Supplementary Online Content

Aoyama H, Uchida K, Aoyama K, et al. Assessment of therapeutic interventions and lung protective ventilation in patients with moderate to severe acute respiratory distress syndrome: a systematic review and network meta-analysis. *JAMA Netw Open*. 2019;2(7):e198116. doi:10.1001/jamanetworkopen.2019.8116

eAppendix 1. Summary of Inclusion and Exclusion Criteria
eAppendix 2. Updated Search Strategy in MEDLINE, Embase, Cochrane, PubMed, and CINAHL
eAppendix 3. Assessment of Heterogeneity, Consistency, and Intransitivity
eAppendix 4. Network Metaregression With Treatment by Covariate Interactions to Measure the Impact of Age and ARDS Severity

eFigure 1. Results of Network Metaregression for Age (a) and ARDS Severity (b)
eFigure 2. Forest Plots of Direct, Indirect, and Pooled Comparisons for Primary Outcome
eFigure 3. Forest Plots of Direct, Indirect, and Pooled Comparisons for Secondary Outcome
eFigure 4. Risk-of-Bias Graph for All Eligible Studies
eFigure 5. Risk-of-Bias Summary for All Eligible Studies
eFigure 6. Funnel Plots for Primary (a) and (b) Secondary Outcomes
eFigure 7. Gelman Plots for Model Convergence With 4 Chains and 2 000 000 Iterated Simulations, Discarding the Initial 1 500 000 Iterations as Burn-in on Primary (a) and (b) Secondary Outcomes
eFigure 8. Ranking Probabilities for the Association of Interventions With Outcomes

eTable 1. Detailed Explanation for Excluded Studies With Full-Text Assessment
eTable 2. Comparison of the Included Interventions in Risk Ratio (95% CrI) for 28-Day Mortality
eTable 3. Network Meta-analysis for 28-Day Mortality and Quality of Evidence Assessment
eTable 4. Results of Sensitivity Analysis for 28-Day Mortality
eTable 5. Results of Preliminary Analysis Excluding Studies Without Description of Cointerventions
eTable 6. Results of Analysis Using Poisson Models for the Primary Outcome Adjusting for Different Timing of the Measurement
eTable 7. Potential Scale Reduction Factor From Brooks-Gelman-Rubin Diagnostic for Model Convergence on Primary (a) and (b) Secondary Outcomes
eTable 8. Assessment of Model Fits for Primary (a) and (b) Secondary Outcomes
eTable 9. Results of Assessment on Small Sample Size Effects
eTable 10. Comparison of the Included Interventions in Risk Ratio (95% CrI) for Barotrauma
eTable 11. Network Meta-analysis for Barotrauma and Quality of Evidence Assessment
eTable 12. Results of Node-Splitting Models
eTable 13. Summary of Cointerventions in Included Studies

eReferences

This supplementary material has been provided by the authors to give readers additional information about their work.

© 2019 Aoyama H et al. *JAMA Network Open.*

eAppendix 1. Summary of Inclusion and Exclusion Criteria

Inclusion Criteria

- Age of patients in study > 18 years
- Patients must have moderate or severe ARDS (Berlin Definition, or $PaO_2/FiO_2 \leq 200$) on mechanical ventilation
- Study setting is ICUs
- Study design is randomized or quasi-randomized clinical trials
- Intervention and control arm – lung protective ventilation alone, or lung protective ventilation and an adjunctive intervention [e.g. prone positioning, recruitment maneuvers (RMs) using CPAP, RM using high PEEP, neuromuscular blockade (NMBA), inhaled nitric oxide (iNO)] or venovenous extracorporeal membrane oxygenation (VV ECMO) or high frequency oscillation ventilation (HFOV)
- Co-interventions in either arm – each arm can have more than one intervention simultaneously
- 28-day mortality. If 28-day mortality is not reported, hospital mortality or mortality at longest follow-up regardless of the duration of follow-up will be employed

Exclusion Criteria

- Editorials, reviews, abstracts or conference proceedings
- Ineligible study designs
  - Controlled studies, observational cohort studies, case-control studies should be excluded
- No relevant population or study setting
  - Age < 18 years
  - Do not meet criteria for moderate or severe ARDS
  - Operating Rooms, Emergency Rooms, PICU and NICU
- No relevant intervention or outcome
  - Irrelevant intervention (i.e. intervention not specified above) should be excluded
  - Mortality not reported
- Animal studies

© 2019 Aoyama H et al. *JAMA Network Open.*

eAppendix 2. Updated Search Strategy in MEDLINE, Embase, Cochrane, PubMed, CINAHL

Medline

Ovid MEDLINE(R) 1946 to May 29, 2019

#	Searches	Results
1	Respiratory Distress Syndrome, Adult/	18172
2	acute respiratory distress syndrome?.mp,kw.	9900
3	adult respiratory distress syndrome?.mp,kw.	4110
4	ARDS.mp,kw.	9541
5	ARDSS.mp,kw.	4
6	shock lung.mp,kw.	402
7	Acute Chest Syndrome/ [New MeSH as of 2010; related to ARDS]	236
8	(acute adj1 chest adj1 syndrome?).mp,kw.	887
9	exp ventilator-induced lung injury/	5179
10	(ventilat* adj1 induced).mp,kw.	2322
11	(acute adj1 lung? adj1 fail*).mp,kw.	114
12	(acute adj1 pulmonary adj1 fail*).mp,kw.	98
13	(acute adj1 bronchopulmonary adj1 fail*).mp,kw.	0
14	(acute adj1 respirat* adj1 fail*).mp,kw.	5569
15	(acute adj1 lung? adj1 injur*).mp,kw.	12571
16	(acute adj1 pulmonary adj1 injur*).mp,kw.	122
17	(acute adj1 bronchopulmonary adj1 injur*).mp,kw.	0
18	(acute adj1 respirat* adj1 injur*).mp,kw.	388
19	Lung Injury/	6322
20	(lung? adj2 injur*).mp,kw.	29126
21	Respiratory Insufficiency/	30784
22	(respirat* adj2 insufficien*).mp,kw.	34404
23	(pulmon* adj2 insufficien*).mp,kw.	3426
24	(bronchopulmon* adj2 insufficien*).mp,kw.	9
25	(cardiopulmon* adj2 insufficien*).mp,kw.	195
26	ALI.ti,ab. [Acute Lung Injury]	5153
27	or/1-26 [ARDS & related terms]	89592
28	(high* adj4 pressur*).mp,kw.	250427
29	(less*5 adj4 pressur*).mp,kw.	9115
30	(low*5 adj4 pressur*).mp,kw.	53025
31	(differen* adj4 pressur*).mp,kw.	21225
32	(variab* adj4 pressur*).mp,kw.	7826
33	(varying adj4 pressur*).mp,kw.	1119
34	Pressure/	70693
35	Lung Compliance/	6425
36	(lung? adj1 complianc*).mp,kw.	7850
37	Tidal Volume/	9541
38	Vt.ti,ab. ["tidal volume"]	13278
39	(tidal adj1 volume?).mp,kw.	17247
40	LVTT.ti,ab. [low volume tidal ventilation]	0
41	(peak adj1 pressure?).mp,kw.	3193
42	(plateau adj1 pressure?).mp,kw.	636
43	(positive adj end-expiratory pressure?).mp,kw.	5093
44	PEEP.mp,kw.	4683
45	exp Respiration, Artificial/	72916
46	(art#ficial* adj2 respirat*).mp,kw.	47072
47	exp Pulmonary Ventilation/	42163
48	(ventilat* adj6 strateg*).mp,kw.	2417
49	exp Ventilators, Mechanical/	8866
50	(mechanic* adj2 ventila*).mp.	48895
51	Prone Position/	3811
52	Pronation/ and ("1988" or "1989" or "1990" or "1991").yr. [historical]	161

© 2019 Aoyama H et al. *JAMA Network Open.*

53	prone.mp.	60289			
54	exp positive-pressure respiration/	24679			
55	(high frequency adj3 ventil*).mp.	4164			
56	HFOV.mp.	642			
57	high-frequency oscillation?.mp.	1508			
58	exp Neuromuscular Blocking Agents/	24337			
59	(neuromuscular adj2 block*).mp.	10345			
60	NMBA.mp.	303			
61	NMBAs.mp.	157			
62	Extracorporeal Membrane Oxygenation/	9154			
63	Extracorporeal Circulation/ and Oxygen/	786			
64	extracorporeal life support*.mp.	1511			
65	extra-corporeal life support*.mp.	41			
66	extracorporeal membrane oxygenat*.mp.	10815			
67	extra-corporeal membrane oxygenat*.mp.	235			
68	ecmo.mp.	5458			
69	ecls.mp.	943			
70	(extracorporeal* adj2 circulation).mp.	15963			
71	(extra-corporeal* adj2 circulation).mp.	296			
72	(extracorporeal adj2 oxygenat*).mp.	11020			
73	(extra-corporeal adj2 oxygenat*).mp.	249			
74	(extracorporeal adj2 support*).mp.	2651			
75	(extra-corporeal adj2 support*).mp.	62			
76	Oxygenators, Membrane/	1694			
77	(membrane? adj2 oxygenat*).mp.	12614			
78	Heart-Lung Machine/	2081			
79	(extracorporeal adj2 lung? adj1 assist*).mp.	183			
80	ECLA.mp.	111			
81	pECLA.mp.	25			
82	Nitric Oxide/ and inhal*.mp.	4307			
83	(Nitric Oxide? and inhal*).mp.	5763			
84	(nitrogen monoxide? and inhal*).mp.	32			
85	(nitrogen oxide? and inhal*).mp.	237			
86	(endogenous adj1 nitrat* adj1 vasodilat*).mp.	2			
87	(mononitro* adj1 monoxide*).mp.	0			
88	(mono-nitro* adj1 monoxide*).mp.	0			
89	iNO.ti,ab.	1390			
90	"inhal* NO".ti,ab.	928			
91	(recruitment adj2 (manoeuv* or maneuv*)).mp.	780			
92	(open adj1 lung?).mp.	2413			
93	(protect* adj2 ventilat*).mp.	1364			
94	(lung? adj2 recruit*).mp.	1089			
95	(alveolar adj2 recruit*).mp.	572			
96	(ART adj strateg*).mp.	141			
97	ARDSnet.mp.	89			
98	or/28-97 [Ventilation Strategies & related terms]	649910			
99	Randomized controlled trial.pt.	482591			
100	exp Randomized controlled trial/	483139			
101	exp Randomized Controlled Trials as Topic/	126565			
102	Pragmatic Clinical Trial.pt.	1060			
103	Pragmatic Clinical Trial/	1060			
104	Pragmatic Clinical Trials As Topic/	304			
105	(pragmatic adj2 (trial? or study or studies)).mp,kw.	2763			
106	random*.mp,kw.	1118669			
107	Double-Blind Method/	151495			
108	((single or double or triple or treble) adj3 (blind* or mask*)).mp,kw.	210907			
109	doubleblind*.mp,kw.	202			
110	Placebos/	34358			
111	Placebo*.mp,kw.	199627			

© 2019 Aoyama H et al. *JAMA Network Open.*

112	or/99-111 [RCTs & related terms]	1201101			
113	27 and 98 and 112 [ARDS + Ventilation Strategies + RCTs]	4087			
114	exp animals/ not (exp animals/ and humans/)	4584326			
115	113 not 114	2961			
116	limit 113 to human	2957			
117	115 or 116	2961			
118	limit 117 to ("all infant (birth to 23 months)" or "all child (0 to 18 years)" or "newborn infant (birth to 1 month)" or "infant (1 to 23 months)" or "preschool child (2 to 5 years)" or "child (6 to 12 years)" or "adolescent (13 to 18 years)")	996			
119	117 not 118	1965			
120	limit 117 to ("all adult (19 plus years)" or "young adult (19 to 24 years)" or "adult (19 to 44 years)" or "young adult and adult (19-24 and 19-44)" or "middle age (45 to 64 years)" or "middle aged (45 plus years)" or "all aged (65 and over)" or "aged (80 and over)")	1430			
121	119 or 120	2153			
122	remove duplicates from 121	2141			
123	limit 122 to dt=20180730-20190530	20			
124	limit 122 to ez=20180730-20190530	20			
125	123 or 124	20			

Medline In-Process

Ovid MEDLINE(R) Epub Ahead of Print and In-Process & Other Non-Indexed Citations May 29, 2019

#	Searches	Results			
1	Respiratory Distress Syndrome, Adult/	0			
2	acute respiratory distress syndrome?.mp,kw.	1990			
3	adult respiratory distress syndrome?.mp,kw.	116			
4	ARDS.mp,kw.	1449			
5	ARDSS.mp,kw.	2			
6	shock lung.mp,kw.	3			
7	Acute Chest Syndrome/ [New MeSH as of 2010; related to ARDS]	0			
8	(acute adj1 chest adj1 syndrome?).mp,kw.	115			
9	exp ventilator-induced lung injury/	0			
10	(ventilat* adj1 induced).mp,kw.	321			
11	(acute adj1 lung? adj1 fail*).mp,kw.	15			
12	(acute adj1 pulmonary adj1 fail*).mp,kw.	6			
13	(acute adj1 bronchopulmonary adj1 fail*).mp,kw.	0			
14	(acute adj1 respirat* adj1 fail*).mp,kw.	788			
15	(acute adj1 lung? adj1 injur*).mp,kw.	1275			
16	(acute adj1 pulmonary adj1 injur*).mp,kw.	10			
17	(acute adj1 bronchopulmonary adj1 injur*).mp,kw.	0			
18	(acute adj1 respirat* adj1 injur*).mp,kw.	48			
19	Lung Injury/	0			
20	(lung? adj2 injur*).mp,kw.	2817			
21	Respiratory Insufficiency/	0			
22	(respirat* adj2 insufficien*).mp,kw.	490			
23	(pulmon* adj2 insufficien*).mp,kw.	120			
24	(bronchopulmon* adj2 insufficien*).mp,kw.	1			
25	(cardiopulmon* adj2 insufficien*).mp,kw.	8			
26	ALI.ti,ab. [Acute Lung Injury]	1106			
27	or/1-26 [ARDS & related terms]	6533			
28	(high* adj4 pressur*).mp,kw.	16842			
29	(less*5 adj4 pressur*).mp,kw.	668			
30	(low*5 adj4 pressur*).mp,kw.	8915			
31	(differen* adj4 pressur*).mp,kw.	3642			
32	(variab* adj4 pressur*).mp,kw.	1066			
33	(varying adj4 pressur*).mp,kw.	324			
34	Pressure/	0			

© 2019 Aoyama H et al. *JAMA Network Open.*

35	Lung Compliance/	0			
36	(lung? adj1 complianc*).mp,kw.	195			
37	Tidal Volume/	0			
38	Vt.ti,ab. ["tidal volume"]	1693			
39	(tidal adj1 volume?).mp,kw.	978			
40	LTVV.ti,ab. [low tidal volume ventilation]	2			
41	(peak adj1 pressure?).mp,kw.	417			
42	(plateau adj1 pressure?).mp,kw.	120			
43	(positive adj end-expiratory pressure?).mp,kw.	522			
44	PEEP.mp,kw.	443			
45	exp Respiration, Artificial/	0			
46	(art#ficial* adj2 respirat*).mp,kw.	185			
47	exp Pulmonary Ventilation/	0			
48	(ventilat* adj6 strateg*).mp,kw.	383			
49	exp Ventilators, Mechanical/	0			
50	(mechanic* adj2 ventila*).mp.	5832			
51	Prone Position/	0			
52	Pronation/ and ("1988" or "1989" or "1990" or "1991").yr. [historical]	0			
53	prone.mp.	9987			
54	exp positive-pressure respiration/	0			
55	(high frequency adj3 ventil*).mp.	211			
56	HFOV.mp.	64			
57	high-frequency oscillation?.mp.	249			
58	exp Neuromuscular Blocking Agents/	0			
59	(neuromuscular adj2 block*).mp.	760			
60	NMBA.mp.	48			
61	NMBAs.mp.	32			
62	Extracorporeal Membrane Oxygenation/	0			
63	Extracorporeal Circulation/ and Oxygen/	0			
64	extracorporeal life support*.mp.	332			
65	extra-corporeal life support*.mp.	10			
66	extracorporeal membrane oxygenat*.mp.	1746			
67	extra-corporeal membrane oxygenat*.mp.	65			
68	ecmo.mp.	1217			
69	ecls.mp.	229			
70	(extracorporeal* adj2 circulation).mp.	311			
71	(extra-corporeal* adj2 circulation).mp.	13			
72	(extracorporeal adj2 oxygenat*).mp.	1777			
73	(extra-corporeal adj2 oxygenat*).mp.	68			
74	(extracorporeal adj2 support*).mp.	506			
75	(extra-corporeal adj2 support*).mp.	11			
76	Oxygenators, Membrane/	0			
77	(membrane? adj2 oxygenat*).mp.	1826			
78	Heart-Lung Machine/	0			
79	(extracorporeal adj2 lung? adj1 assist*).mp.	12			
80	ECLA.mp.	7			
81	pECLA.mp.	4			
82	Nitric Oxide/ and inhal*.mp.	0			
83	(Nitric Oxide? and inhal*).mp.	382			
84	(nitrogen monoxide? and inhal*).mp.	4			
85	(nitrogen oxide? and inhal*).mp.	9			
86	(endogenous adj1 nitrat* adj1 vasodilat*).mp.	0			
87	(mononitro* adj1 monoxide*).mp.	0			
88	(mono-nitro* adj1 monoxide*).mp.	0			
89	iNO.ti,ab.	234			
90	"inhal* NO".ti,ab.	37			
91	(recruitment adj2 (manoeuv* or maneuv*)).mp.	131			
92	(open adj1 lung?).mp.	117			
93	(protect* adj2 ventilat*).mp.	311			

© 2019 Aoyama H et al. *JAMA Network Open.*

94	(lung? adj2 recruit*).mp.	114			
95	(alveolar adj2 recruit*).mp.	60			
96	(ART adj strateg*).mp.	44			
97	(low?? adj1 airway? adj1 pressure?).mp.	11			
98	ARDSNet.mp.	13			
99	or/28-98 [Ventilation Strategies & related terms]	49790			
100	Randomized controlled trial.pt.	277			
101	exp Randomized controlled trial/	278			
102	exp Randomized Controlled Trials as Topic/	0			
103	Pragmatic Clinical Trial.pt.	0			
104	Pragmatic Clinical Trial/	0			
105	Pragmatic Clinical Trials As Topic/	0			
106	(pragmatic adj2 (trial? or study or studies)).mp,kw.	482			
107	random*.mp,kw.	156957			
108	Double-Blind Method/	0			
109	((single or double or triple or treble) adj3 (blind* or mask*)).mp,kw.	15486			
110	doubleblind*.mp,kw.	25			
111	Placebos/	0			
112	Placebo*.mp,kw.	19013			
113	or/100-112 [RCTs & related terms]	164072			
114	27 and 99 and 113 [ARDS + Ventilation Strategies + RCTs]	293			
115	remove duplicates from 114	292			
116	limit 115 to dt=20180730-20190530	100			
117	limit 115 to ez=20180730-20190530	85			
118	116 or 117 [Update Citations]	100			

Embase

Embase Classic+Embase 1947 to 2019 May 29

#	Searches	Results			
1	Respiratory Distress Syndrome, Adult/	13327			
2	acute respiratory distress syndrome?.mp,kw.	18101			
3	adult respiratory distress syndrome?.mp,kw.	34798			
4	ARDS.mp,kw.	19110			
5	ARDSS.mp,kw.	9			
6	shock lung.mp,kw.	929			
7	Acute Chest Syndrome/ [New MeSH as of 2010; related to ARDS]	2066			
8	(acute adj1 chest adj1 syndrome?).mp,kw.	2481			
9	exp ventilator-induced lung injury/	2021			
10	(ventilat* adj1 induced).mp,kw.	4235			
11	(acute adj1 lung? adj1 fail*).mp,kw.	183			
12	(acute adj1 pulmonary adj1 fail*).mp,kw.	167			
13	(acute adj1 bronchopulmonary adj1 fail*).mp,kw.	0			
14	(acute adj1 respirat* adj1 fail*).mp,kw.	15023			
15	(acute adj1 lung? adj1 injur*).mp,kw.	22751			
16	(acute adj1 pulmonary adj1 injur*).mp,kw.	179			
17	(acute adj1 bronchopulmonary adj1 injur*).mp,kw.	0			
18	(acute adj1 respirat* adj1 injur*).mp,kw.	580			
19	Lung Injury/	34293			
20	(lung? adj2 injur*).mp,kw.	61190			
21	Respiratory Insufficiency/	30255			
22	(respirat* adj2 insufficien*).mp,kw.	13658			
23	(pulmon* adj2 insufficien*).mp,kw.	6738			
24	(bronchopulmon* adj2 insufficien*).mp,kw.	16			
25	(cardiopulmon* adj2 insufficien*).mp,kw.	5121			
26	adult respiratory distress syndrome/ [Embase]	33050			
27	respiratory distress syndrome/ [Embase]	13989			

© 2019 Aoyama H et al. *JAMA Network Open.*

28	acute chest syndrome/ [Embase]	2066			
29	lung injury/ [Embase]	34293			
30	lung insufficiency/ [Embase]	5083			
31	acute respiratory failure/ [Embase]	11365			
32	respiratory failure/ [Embase]	69181			
33	cardiopulmonary insufficiency/ [Embase]	4911			
34	ALI.ti,ab. [Acute Lung Injury]	11367			
35	or/1-34 [ARDS & related terms]	201812			
36	(high* adj4 pressur*).mp,kw.	122482			
37	(less*5 adj4 pressur*).mp,kw.	9281			
38	(low*5 adj4 pressur*).mp,kw.	86522			
39	(differen* adj4 pressur*).mp,kw.	33851			
40	(variab* adj4 pressur*).mp,kw.	14235			
41	(varying adj4 pressur*).mp,kw.	1844			
42	Pressure/	59231			
43	Lung Compliance/	12116			
44	(lung? adj1 complianc*).mp,kw.	13216			
45	Tidal Volume/	21131			
46	Vt.ti,ab. ["tidal volume"]	26318			
47	(tidal adj1 volume?).mp,kw.	27522			
48	LVTT.ti,ab. [low volume tidal ventilation]	0			
49	(peak adj1 pressure?).mp,kw.	5047			
50	(plateau adj1 pressure?).mp,kw.	1332			
51	(positive adj end-expiratory pressure?).mp,kw.	54133			
52	PEEP.mp,kw.	8748			
53	exp Respiration, Artificial/	195242			
54	(art#ficial* adj2 respirat*).mp,kw.	4479			
55	exp Pulmonary Ventilation/	37570			
56	(ventilat* adj6 strateg*).mp,kw.	4295			
57	exp Ventilators, Mechanical/	2963			
58	(mechanic* adj2 ventila*).mp.	79482			
59	Prone Position/	1872			
60	Pronation/	653			
61	prone.mp.	96805			
62	exp positive-pressure respiration/	52912			
63	(high frequency adj3 ventil*).mp.	5990			
64	HFOV.mp.	1110			
65	high-frequency oscillation?.mp.	4146			
66	exp Neuromuscular Blocking Agents/	77170			
67	(neuromuscular adj2 block*).mp.	21864			
68	NMBA.mp.	550			
69	NMBAs.mp.	279			
70	Extracorporeal Membrane Oxygenation/	20361			
71	Extracorporeal Circulation/ and Oxygen/	980			
72	extracorporeal life support*.mp.	2885			
73	extra-corporeal life support*.mp.	145			
74	extracorporeal membrane oxygenat*.mp.	14484			
75	extra-corporeal membrane oxygenat*.mp.	815			
76	ecmo.mp.	13857			
77	ecls.mp.	1983			
78	(extracorporeal* adj2 circulation).mp.	27006			
79	(extra-corporeal* adj2 circulation).mp.	539			
80	(extracorporeal adj2 oxygenat*).mp.	22528			
81	(extra-corporeal adj2 oxygenat*).mp.	859			
82	(extracorporeal adj2 support*).mp.	4806			
83	(extra-corporeal adj2 support*).mp.	197			
84	Oxygenators, Membrane/	562			
85	(membrane? adj2 oxygenat*).mp.	17419			
86	Heart-Lung Machine/	2426			

© 2019 Aoyama H et al. *JAMA Network Open.*

87	(extracorporeal adj2 lung? adj1 assist*).mp.	280			
88	ECLA.mp.	191			
89	pECLA.mp.	55			
90	Nitric Oxide/ and inhal*.mp.	10584			
91	(Nitric Oxide? and inhal*).mp.	12189			
92	(nitrogen monoxide? and inhal*).mp.	47			
93	(nitrogen oxide? and inhal*).mp.	450			
94	(endogenous adj1 nitrat* adj1 vasodilat*).mp.	2			
95	(mononitro* adj1 monoxide*).mp.	0			
96	(mono-nitro* adj1 monoxide*).mp.	0			
97	iNO.ti,ab.	2480			
98	"inhal* NO".ti,ab.	1246			
99	(recruitment adj2 (manoeuv* or maneuv*)).mp.	1512			
100	(open adj1 lung?).mp.	4237			
101	(protect* adj2 ventilat*).mp.	2785			
102	(lung? adj2 recruit*).mp.	1976			
103	(alveolar adj2 recruit*).mp.	981			
104	(ART adj strateg*).mp.	266			
105	ARDSNet.mp.	315			
106	airway pressure/ [Embase]	10673			
107	artificial ventilation/ [Embase]	128831			
108	extracorporeal membrane oxygenation device/ [Embase]	1202			
109	extracorporeal oxygenation/ [Embase]	20361			
110	exp high frequency ventilation/ [Embase]	3840			
111	lung pressure/ [Embase]	6293			
112	lung wedge pressure/ [Embase]	3818			
113	exp lung ventilation/ [Embase]	37570			
114	membrane oxygenator/ [Embase]	562			
115	exp neuromuscular blocking agent/ [Embase]	77170			
116	positive end expiratory pressure/ [Embase]	52912			
117	positive end expiratory pressure ventilator/ [Embase]	285			
118	pressure support ventilation/ [Embase]	1454			
119	or/36-118 [Ventilation Strategies & related terms]	798973			
120	35 and 119 [ARDS + Care Strategies]	64322			
121	exp Randomized controlled trial/	553945			
122	exp Randomized Controlled Trials as Topic/	160716			
123	Pragmatic Clinical Trial/	435			
124	Pragmatic Clinical Trials As Topic/	160716			
125	(pragmatic adj2 (trial? or study or studies)).mp,kw.	3275			
126	random*.mp,kw.	1637980			
127	Double-Blind Method/	129211			
128	((single or double or triple or treble) adj3 (blind* or mask*)).mp,kw.	295619			
129	doubleblind*.mp,kw.	3180			
130	Placebos/	288201			
131	Placebo*.mp,kw.	443158			
132	"Randomized Controlled Trial (Topic)"/ [Embase]	160716			
133	placebo/ [Embase]	345119			
134	placebo effect/ [Embase]	5503			
135	double blind procedure/ [Embase]	163420			
136	randomization/ [Embase]	82764			
137	or/121-136 [RCTs]	1880130			
138	120 and 137 [ARDS + Care Strategies + RCTs]	7817			
139	(exp animals/ or exp animal experimentation/ or nonhuman/) not ((exp animals/ or exp animal experimentation/ or nonhuman/) and exp human/)	6991452			
140	138 not 139	6187			
141	limit 138 to human	5923			
142	140 or 141	6187			
143	limit 142 to (embryo <first trimester> or infant <to one year> or child <unspecified age> or preschool child <1 to 6 years> or school child <7 to 12 years> or adolescent <13 to 17 years>)	547			

© 2019 Aoyama H et al. *JAMA Network Open.*

144	142 not 143	5640			
145	limit 142 to (adult <18 to 64 years> or aged <65+ years>)	1960			
146	144 or 145	5778			
147	limit 146 to conference abstract status	1071			
148	limit 146 to (books or "book review" or chapter or conference abstract or "conference review")	1073			
149	limit 146 to (book or book series or conference proceeding or trade journal)	8			
150	147 or 148 or 149	1079			
151	146 not 150	4699			
152	remove duplicates from 151	4609			
153	limit 152 to dc=20180730-20190530	254			
154	"201832".em.	33372			
155	"201833".em.	48811			
156	"201834".em.	26112			
157	"201835".em.	33602			
158	"201836".em.	31182			
159	"201837".em.	32687			
160	"201838".em.	29511			
161	"201839".em.	30727			
162	"20184#".em.	340456			
163	"20185#".em.	123269			
164	or/154-163	729729			
165	152 and 164	130			
166	limit 152 to yr="2019 -Current"	121			
167	165 or 166	249			
168	153 or 166	254			

CCTR

Cochrane Central Register of Controlled Trials 2014 to Present

#	Searches	Results			
1	Respiratory Distress Syndrome, Adult/	1022			
2	acute respiratory distress syndrome?.mp,kw.	1328			
3	adult respiratory distress syndrome?.mp,kw.	1156			
4	ARDS.mp,kw.	1445			
5	ARDSS.mp,kw.	0			
6	shock lung.mp,kw.	10			
7	Acute Chest Syndrome/ [New MeSH as of 2010; related to ARDS]	24			
8	(acute adj1 chest adj1 syndrome?).mp,kw.	169			
9	exp ventilator-induced lung injury/	810			
10	(ventilat* adj1 induced).mp,kw.	202			
11	(acute adj1 lung? adj1 fail*).mp,kw.	13			
12	(acute adj1 pulmonary adj1 fail*).mp,kw.	9			
13	(acute adj1 bronchopulmonary adj1 fail*).mp,kw.	0			
14	(acute adj1 respirat* adj1 fail*).mp,kw.	1037			
15	(acute adj1 lung? adj1 injur*).mp,kw.	1002			
16	(acute adj1 pulmonary adj1 injur*).mp,kw.	3			
17	(acute adj1 bronchopulmonary adj1 injur*).mp,kw.	0			
18	(acute adj1 respirat* adj1 injur*).mp,kw.	124			
19	Lung Injury/	169			
20	(lung? adj2 injur*).mp,kw.	2102			
21	Respiratory Insufficiency/	1237			
22	(respirat* adj2 insufficien*).mp,kw.	1562			
23	(pulmon* adj2 insufficien*).mp,kw.	204			
24	(bronchopulmon* adj2 insufficien*).mp,kw.	2			
25	(cardiopulmon* adj2 insufficien*).mp,kw.	149			
26	adult respiratory distress syndrome/ [Embase]	1022			
27	respiratory distress syndrome/ [Embase]	0			
28	acute chest syndrome/ [Embase]	24			

© 2019 Aoyama H et al. *JAMA Network Open.*

29	lung injury/ [Embase]	169			
30	lung insufficiency/ [Embase]	0			
31	acute respiratory failure/ [Embase]	0			
32	respiratory failure/ [Embase]	1237			
33	cardiopulmonary insufficiency/ [Embase]	0			
34	ALI.ti,ab. [Acute Lung Injury]	588			
35	or/1-34 [ARDS & related terms]	7313			
36	(high* adj4 pressur*).mp,kw.	11792			
37	(less*5 adj4 pressur*).mp,kw.	3095			
38	(low*5 adj4 pressur*).mp,kw.	13419			
39	(differen* adj4 pressur*).mp,kw.	7173			
40	(variab* adj4 pressur*).mp,kw.	2191			
41	(varying adj4 pressur*).mp,kw.	84			
42	Pressure/	2463			
43	Lung Compliance/	248			
44	(lung? adj1 complianc*).mp,kw.	701			
45	Tidal Volume/	825			
46	Vt.ti,ab. ["tidal volume"]	1960			
47	(tidal adj1 volume?).mp,kw.	3350			
48	LVTT.ti,ab. [low volume tidal ventilation]	0			
49	(peak adj1 pressure?).mp,kw.	561			
50	(plateau adj1 pressure?).mp,kw.	257			
51	(positive adj end-expiratory pressure?).mp,kw.	3521			
52	PEEP.mp,kw.	1421			
53	exp Respiration, Artificial/	5608			
54	(art#ficial* adj2 respirat*).mp,kw.	3151			
55	exp Pulmonary Ventilation/	6927			
56	(ventilat* adj6 strateg*).mp,kw.	771			
57	exp Ventilators, Mechanical/	254			
58	(mechanic* adj2 ventila*).mp.	9651			
59	Prone Position/	281			
60	Pronation/	64			
61	prone.mp.	3797			
62	exp positive-pressure respiration/	2479			
63	(high frequency adj3 ventil*).mp.	562			
64	HFOV.mp.	145			
65	high-frequency oscillation?.mp.	183			
66	exp Neuromuscular Blocking Agents/	2199			
67	(neuromuscular adj2 block*).mp.	3113			
68	NMBA.mp.	74			
69	NMBAs.mp.	38			
70	Extracorporeal Membrane Oxygenation/	154			
71	Extracorporeal Circulation/ and Oxygen/	19			
72	extracorporeal life support*.mp.	53			
73	extra-corporeal life support*.mp.	2			
74	extracorporeal membrane oxygenat*.mp.	522			
75	extra-corporeal membrane oxygenat*.mp.	36			
76	ecmo.mp.	437			
77	ecls.mp.	43			
78	(extracorporeal* adj2 circulation).mp.	1089			
79	(extra-corporeal* adj2 circulation).mp.	30			
80	(extracorporeal adj2 oxygenat*).mp.	656			
81	(extra-corporeal adj2 oxygenat*).mp.	36			
82	(extracorporeal adj2 support*).mp.	136			
83	(extra-corporeal adj2 support*).mp.	2			
84	Oxygenators, Membrane/	79			
85	(membrane? adj2 oxygenat*).mp.	694			
86	Heart-Lung Machine/	35			
87	(extracorporeal adj2 lung? adj1 assist*).mp.	9			

© 2019 Aoyama H et al. *JAMA Network Open.*

88	ECLA.mp.	18			
89	pECLA.mp.	2			
90	Nitric Oxide/ and inhal*.mp.	548			
91	(Nitric Oxide? and inhal*).mp.	1503			
92	(nitrogen monoxide? and inhal*).mp.	4			
93	(nitrogen oxide? and inhal*).mp.	9			
94	(endogenous adj1 nitrat* adj1 vasodilat*).mp.	0			
95	(mononitro* adj1 monoxide*).mp.	0			
96	(mono-nitro* adj1 monoxide*).mp.	0			
97	iNO.ti,ab.	326			
98	"inhal* NO".ti,ab.	26460			
99	(recruitment adj2 (manoeuv* or maneuv*)).mp.	427			
100	(open adj1 lung?).mp.	134			
101	(protect* adj2 ventilat*).mp.	428			
102	(lung? adj2 recruit*).mp.	345			
103	(alveolar adj2 recruit*).mp.	202			
104	(ART adj strateg*).mp.	46			
105	ARDSNet.mp.	66			
106	airway pressure/ [Embase]	945			
107	artificial ventilation/ [Embase]	1			
108	extracorporeal membrane oxygenation device/ [Embase]	0			
109	extracorporeal oxygenation/ [Embase]	0			
110	exp high frequency ventilation/ [Embase]	182			
111	lung pressure/ [Embase]	0			
112	lung wedge pressure/ [Embase]	0			
113	membrane oxygenator/ [Embase]	79			
114	positive end expiratory pressure/ [Embase]	1			
115	positive end expiratory pressure ventilator/ [Embase]	0			
116	pressure support ventilation/ [Embase]	0			
117	or/36-116 [Care Strategies]	87702			
118	35 and 117 [ARDS + Care Strategies]	3890			
119	randomized controlled trial/ [Medline/Embase]	129			
120	"Randomized Controlled Trial (Topic)"/ [Embase]	6			
121	randomized controlled trials as topic/ [Medline]	5838			
122	random*.mp.	943567			
123	or/119-122 [RCTs]	943567			
124	118 and 123 [ARDS + Care Strategies + RCTs]	3007			
125	conference*.so.	42881			
126	congress*.so.	14818			
127	poster?.so.	14			
128	limit 118 to (bibliography or conference or congresses or monograph)	39			
129	or/125-128	43267			
130	124 not 129	2902			
131	(paedia* or child* or infant* or neonat* or pediatr* or teen*).jw.	32947			
132	(paedia* or child* or infant* or neonat* or pediatr* or teen*).ti.	98812			
133	or/131-132	111621			
134	130 not 133	2356			
135	remove duplicates from 134	2286			
136	limit 135 to latest update	44			

CDSR

Cochrane Database of Systematic Reviews 2005 to Present

#	Searches	Results
1	acute respiratory distress syndrome?.mp,kw.	103

© 2019 Aoyama H et al. *JAMA Network Open.*

2	adult respiratory distress syndrome?.mp,kw.	26
3	ARDS.mp,kw.	73
4	ARDSS.mp,kw.	0
5	shock lung.mp,kw.	1
6	(acute adj1 chest adj1 syndrome?).mp,kw.	39
7	(ventilat* adj1 induced).mp,kw.	33
8	(acute adj1 lung? adj1 fail*).mp,kw.	3
9	(acute adj1 pulmonary adj1 fail*).mp,kw.	1
10	(acute adj1 bronchopulmonary adj1 fail*).mp,kw.	0
11	(acute adj1 respirat* adj1 fail*).mp,kw.	53
12	(acute adj1 lung? adj1 injur*).mp,kw.	78
13	(acute adj1 pulmonary adj1 injur*).mp,kw.	1
14	(acute adj1 bronchopulmonary adj1 injur*).mp,kw.	0
15	(acute adj1 respirat* adj1 injur*).mp,kw.	2
16	(lung? adj2 injur*).mp,kw.	170
17	(respirat* adj2 insufficien*).mp,kw.	107
18	(pulmon* adj2 insufficien*).mp,kw.	10
19	(bronchopulmon* adj2 insufficien*).mp,kw.	0
20	(cardiopulmon* adj2 insufficien*).mp,kw.	1
21	ALI.ti,ab. [Acute Lung Injury]	4
22	or/1-21 [ARDS]	402
23	(high* adj4 pressur*).mp,kw.	693
24	(less*5 adj4 pressur*).mp,kw.	138
25	(low*5 adj4 pressur*).mp,kw.	639
26	(differen* adj4 pressur*).mp,kw.	282
27	(variab* adj4 pressur*).mp,kw.	68
28	(varying adj4 pressur*).mp,kw.	9
29	(lung? adj1 complianc*).mp,kw.	53
30	Vt.ti,ab. ["tidal volume"]	8
31	(tidal adj1 volume?).mp,kw.	95
32	LVTT.ti,ab. [low volume tidal ventilation]	0
33	(peak adj1 pressure?).mp,kw.	22
34	(plateau adj1 pressure?).mp,kw.	12
35	(positive adj end-expiratory pressure?).mp,kw.	73
36	PEEP.mp,kw.	58
37	(art#ficial* adj2 respirat*).mp,kw.	123
38	(ventilat* adj6 strateg*).mp,kw.	81
39	(mechanic* adj2 ventila*).mp.	596

© 2019 Aoyama H et al. *JAMA Network Open.*

40	prone.mp.	1113
41	(high frequency adj3 ventil*).mp.	66
42	HFOV.mp.	25
43	high-frequency oscillation?.mp.	13
44	(neuromuscular adj2 block*).mp.	57
45	NMBA.mp.	4
46	NMBAs.mp.	4
47	extracorporeal life support*.mp.	8
48	extra-corporeal life support*.mp.	0
49	extracorporeal membrane oxygenat*.mp.	38
50	extra-corporeal membrane oxygenat*.mp.	3
51	ecmo.mp.	36
52	ecls.mp.	2
53	(extracorporeal* adj2 circulation).mp.	13
54	(extra-corporeal* adj2 circulation).mp.	0
55	(extracorporeal adj2 oxygenat*).mp.	39
56	(extra-corporeal adj2 oxygenat*).mp.	3
57	(extracorporeal adj2 support*).mp.	12
58	(extra-corporeal adj2 support*).mp.	0
59	(membrane? adj2 oxygenat*).mp.	40
60	(extracorporeal adj2 lung? adj1 assist*).mp.	1
61	ECLA.mp.	1
62	pECLA.mp.	1
63	(Nitric Oxide? and inhal*).mp.	101
64	(nitrogen monoxide? and inhal*).mp.	1
65	(nitrogen oxide? and inhal*).mp.	1
66	(endogenous adj1 nitrat* adj1 vasodilat*).mp.	0
67	(mononitro* adj1 monoxide*).mp.	0
68	(mono-nitro* adj1 monoxide*).mp.	0
69	iNO.ti,ab.	10
70	"inhal* NO".ti,ab.	1
71	(recruitment adj2 (manoeuv* or maneuv*)).mp.	17
72	(open adj1 lung?).mp.	9
73	(protect* adj2 ventilat*).mp.	21
74	(lung? adj2 recruit*).mp.	20
75	(alveolar adj2 recruit*).mp.	21
76	(ART adj strateg*).mp.	2
77	ARDSNet.mp.	5

© 2019 Aoyama H et al. *JAMA Network Open.*

78	or/23-77 [Care Strategies]	2686
79	22 and 78 [ARDS + Care Strategies]	282
80	limit 79 to full systematic reviews	249
81	(paedia* or child* or infant* or neonat* or pediatr* or teen*).gw.	2628
82	80 not 81	132
83	remove duplicates from 82	132
84	limit 83 to last year	60

© 2019 Aoyama H et al. *JAMA Network Open.*

PubMed-NOT-Medline

Search	Query	Items found
#1	Search (((((((((("Respiratory Distress Syndrome, Adult"[MeSH] OR "Acute Chest Syndrome"[MeSH] OR "Ventilator-Induced Lung Injury"[MeSH] OR "Lung Injury"[MeSH] OR "Respiratory Insufficiency"[MeSH] OR (acute respiratory distress syndrome) OR (adult respiratory distress syndrome) OR ARDS OR ARDSS OR "shock lung" OR (acute chest syndrome) OR (ventilation induced) OR (acute lung failure) OR (acute pulmonary failure) OR (acute bronchopulmonary failure) OR (acute respiratory failure) OR (acute lung injury) OR (acute lung injuries) OR (acute pulmonary injury) OR (acute pulmonary injuries) OR (acute bronchopulmonary injury) OR (acute bronchopulmonary injuries) OR (acute respiratory injury) OR (acute respiratory injuries) OR (respiratory insufficiency) OR (pulmonary insufficiency) OR (bronchopulmonary insufficiency) OR (cardiopulmonary insufficiency) OR "ALI"[tiab])))) AND (((("Pressure"[MeSH] OR "Lung Compliance"[MeSH] OR "Tidal Volume"[MeSH] OR" Respiration, Artificial"[MeSH] OR "Pulmonary Ventilation"[MeSH] OR "Ventilators, Mechanical"[MeSH] OR "Prone Position"[MeSH] OR "positive-pressure respiration"[MeSH] OR "Neuromuscular Blocking Agents"[MeSH] OR "Extracorporeal Membrane Oxygenation"[MeSH] OR ("Extracorporeal Circulation"[MeSH] AND "Oxygen"[MeSH]) OR "Oxygenators, Membrane"[MeSH] OR "Heart-Lung Machine"[MeSH] OR "ARDSnet"[tiab] OR "ECLA"[tiab] OR "ecls"[tiab] OR "ecmo"[tiab] OR "extracorporeal life support" OR "extra-corporeal life support" OR "extracorporeal membrane oxygenation" OR "extra-corporeal membrane oxygenation" OR "HFOV"[tiab] OR "iNO"[tiab] OR "LVTT"[tiab] OR "NMBA"[tiab] OR "NMBAs"[tiab] OR "pECLA"[tiab] OR "PEEP"[tiab] OR ("alveolar recruitment") OR ("ART strategy") OR ("artificial respiration") OR ("different pressure") OR ("endogenous nitrate vasodilation") OR ("extracorporeal lung assistance") OR ("extracorporeal oxygenation") OR ("extra-corporeal oxygenation") OR ("extracorporeal support") OR ("extra-corporeal support") OR ("extracorporeal circulation") OR ("extra-corporeal circulation") OR ("high frequency ventilation") OR ("high pressure") OR ("inhaled Nitric Oxide") OR ("inhaled nitrogen monoxide") OR ("inhaled nitrogen oxide") OR ("less pressure") OR ("low pressure") OR ("lung compliance") OR ("lung recruitment") OR ("mechanical ventilation") OR ("membrane oxygenation") OR ("mononitrogen monoxide") OR ("mono-nitrogen monoxide") OR ("neuromuscular blocking") OR ("open lung") OR ("peak pressure") OR ("plateau pressure") OR ("positive end-expiratory pressure") OR ("protective ventilation") OR ("recruitment maneuver") OR ("recruitment manoeuver") OR ("tidal volume") OR ("variable pressure") OR ("varying pressure") OR ("ventilation strategy") OR "high-frequency oscillation" OR prone))))))) AND (((random OR randomized OR randomised OR randomization OR randomisation OR randomized controlled trial OR randomised controlled trial OR randomized controlled trials OR randomised controlled trials))))))) AND ((((publisher[sb] NOT pubstatusnihms NOT pubstatuspmcsd NOT pmcbook) OR inprocess[sb] OR pubmednotmedline[sb] OR ((pubstatusnihms OR pubstatuspmcsd) AND publisher[sb])))))) AND Sort by: PublicationDate	260
#2	Search (((((((((("Respiratory Distress Syndrome, Adult"[MeSH] OR "Acute Chest Syndrome"[MeSH] OR "Ventilator-Induced Lung Injury"[MeSH] OR "Lung Injury"[MeSH] OR "Respiratory Insufficiency"[MeSH] OR (acute respiratory distress syndrome) OR (adult respiratory distress syndrome) OR ARDS OR ARDSS OR "shock lung" OR (acute chest syndrome) OR (ventilation induced) OR (acute lung failure) OR (acute pulmonary failure) OR (acute bronchopulmonary failure) OR (acute respiratory failure) OR (acute lung injury) OR (acute lung injuries) OR (acute pulmonary injury) OR (acute pulmonary injuries) OR (acute bronchopulmonary injury) OR (acute bronchopulmonary injuries) OR (acute respiratory injury) OR (acute respiratory injuries) OR (respiratory insufficiency) OR (pulmonary insufficiency) OR (bronchopulmonary insufficiency) OR (cardiopulmonary insufficiency) OR "ALI"[tiab])))) AND (((("Pressure"[MeSH] OR "Lung Compliance"[MeSH] OR "Tidal Volume"[MeSH] OR" Respiration, Artificial"[MeSH] OR "Pulmonary Ventilation"[MeSH] OR "Ventilators, Mechanical"[MeSH] OR "Prone Position"[MeSH] OR "positive-pressure respiration"[MeSH] OR "Neuromuscular Blocking Agents"[MeSH] OR "Extracorporeal Membrane Oxygenation"[MeSH] OR ("Extracorporeal Circulation"[MeSH] AND "Oxygen"[MeSH]) OR "Oxygenators, Membrane"[MeSH] OR "Heart-Lung Machine"[MeSH] OR "ARDSnet"[tiab] OR "ECLA"[tiab] OR "ecls"[tiab] OR "ecmo"[tiab] OR "extracorporeal life support" OR "extra-corporeal life support" OR "extracorporeal membrane oxygenation" OR "extra-corporeal membrane oxygenation" OR "HFOV"[tiab] OR "iNO"[tiab] OR "LVTT"[tiab] OR "NMBA"[tiab] OR "NMBAs"[tiab] OR "pECLA"[tiab] OR "PEEP"[tiab] OR ("alveolar recruitment") OR ("ART strategy") OR ("artificial respiration") OR ("different pressure") OR ("endogenous nitrate vasodilation") OR ("extracorporeal lung assistance") OR ("extracorporeal oxygenation") OR ("extra-corporeal oxygenation") OR ("extracorporeal support") OR ("extra-corporeal support") OR ("extracorporeal circulation") OR ("extra-corporeal circulation") OR ("high frequency	93

© 2019 Aoyama H et al. *JAMA Network Open.*

	ventilation") OR ("high pressure") OR ("inhaled Nitric Oxide") OR ("inhaled nitrogen monoxide") OR ("inhaled nitrogen oxide") OR ("less pressure") OR ("low pressure") OR ("lung compliance") OR ("lung recruitment") OR ("mechanical ventilation") OR ("membrane oxygenation") OR ("mononitrogen monoxide") OR ("mono-nitrogen monoxide") OR ("neuromuscular blocking") OR ("open lung") OR ("peak pressure") OR ("plateau pressure") OR ("positive end-expiratory pressure") OR ("protective ventilation") OR ("recruitment maneuver") OR ("recruitment manoeuver") OR ("tidal volume") OR ("variable pressure") OR ("varying pressure") OR ("ventilation strategy") OR "high-frequency oscillation" OR prone))))))) AND (((random OR randomized OR randomised OR randomization OR randomisation OR randomized controlled trial OR randomised controlled trial OR randomized controlled trials OR randomised controlled trials))))))) AND ((((publisher[sb] NOT pubstatusnihms NOT pubstatuspmcsd NOT pmcbook) OR inprocess[sb] OR pubmednotmedline[sb] OR ((pubstatusnihms OR pubstatuspmcsd) AND publisher[sb])))))) AND ("2018/07/30"[Date - Create] : "3000"[Date - Create]))	

CINAHL

ARDS + Care Strategies + RCTs

#	Query	Limiters/Expanders	Last Run Via	Results
S45	S43	Limiters - Published Date: 20180701-20190531<br>Search modes - Boolean/Phrase	Interface - EBSCOhost Research Databases<br>Search Screen - Advanced Search<br>Database - CINAHL with Full Text	6
S44	S43	Search modes - Boolean/Phrase	Interface - EBSCOhost Research Databases<br>Search Screen - Advanced Search<br>Database - CINAHL with Full Text	475
S43	S39 OR S40	Limiters - Peer Reviewed; Publication Type: Journal Article<br>Search modes - Boolean/Phrase	Interface - EBSCOhost Research Databases<br>Search Screen - Advanced Search<br>Database - CINAHL with Full Text	475
S42	S39 OR S40	Limiters - Peer Reviewed<br>Search modes - Boolean/Phrase	Interface - EBSCOhost Research Databases<br>Search Screen - Advanced Search<br>Database - CINAHL with Full Text	506
S41	S39 OR S40	Search modes - Boolean/Phrase	Interface - EBSCOhost Research Databases<br>Search Screen - Advanced Search<br>Database - CINAHL with Full Text	539
S40	S28 AND S37	Limiters - Randomized Controlled Trials; Age Groups: Adult: 19-44 years, Middle Aged: 45-64 years, Aged: 65+ years, Aged, 80 and over, All Adult<br>Search modes - Boolean/Phrase	Interface - EBSCOhost Research Databases<br>Search Screen - Advanced Search<br>Database - CINAHL with Full Text	193
S39	S28 AND S37	Limiters - Human; Age Groups: Adult: 19-44 years, Middle Aged: 45-64 years, Aged: 65+ years, Aged, 80 and over, All Adult<br>Search modes - Boolean/Phrase	Interface - EBSCOhost Research Databases<br>Search Screen - Advanced Search<br>Database - CINAHL with Full Text	538
S38	S28 AND S37	Search modes - Boolean/Phrase	Interface - EBSCOhost Research Databases<br>Search Screen - Advanced Search<br>Database - CINAHL with Full Text	1,703
S37	S29 OR S30 OR S31 OR S32 OR S33 OR S34 OR S35 OR S36	Search modes - Boolean/Phrase	Interface - EBSCOhost Research Databases<br>Search Screen - Advanced Search<br>Database - CINAHL with Full Text	373,884
S36	(pragmatic W2 (trial* or study or studies))	Search modes - Boolean/Phrase	Interface - EBSCOhost Research Databases<br>Search Screen - Advanced Search<br>Database - CINAHL with Full Text	1,823
S35	Placebo*	Search modes - Boolean/Phrase	Interface - EBSCOhost Research Databases<br>Search Screen - Advanced Search<br>Database - CINAHL with Full Text	55,231
S34	(MH "Placebos") OR (MH "Placebo Effect")	Search modes - Boolean/Phrase	Interface - EBSCOhost Research Databases<br>Search Screen - Advanced Search<br>Database - CINAHL with Full Text	12,892
S33	doubleblind* AND double-blind*	Search modes - Boolean/Phrase	Interface - EBSCOhost Research Databases<br>Search Screen - Advanced Search<br>Database - CINAHL with Full Text	74

© 2019 Aoyama H et al. *JAMA Network Open.*

S32	((single or double or triple or treble) W3 (blind* or mask*))	Search modes - Boolean/Phrase	Interface - EBSCOhost Research Databases<br>Search Screen - Advanced Search<br>Database - CINAHL with Full Text	65,853
S31	(MH "Double-Blind Studies")	Search modes - Boolean/Phrase	Interface - EBSCOhost Research Databases<br>Search Screen - Advanced Search<br>Database - CINAHL with Full Text	41,372
S30	random*	Search modes - Boolean/Phrase	Interface - EBSCOhost Research Databases<br>Search Screen - Advanced Search<br>Database - CINAHL with Full Text	348,362
S29	(MH "Random Assignment") OR (MH "Randomized Controlled Trials")	Search modes - Boolean/Phrase	Interface - EBSCOhost Research Databases<br>Search Screen - Advanced Search<br>Database - CINAHL with Full Text	121,150
S28	S9 AND S27	Search modes - Boolean/Phrase	Interface - EBSCOhost Research Databases<br>Search Screen - Advanced Search<br>Database - CINAHL with Full Text	10,095
S27	S10 OR S11 OR S12 OR S13 OR S14 OR S15 OR S16 OR S17 OR S18 OR S19 OR S20 OR S21 OR S22 OR S23 OR S24 OR S25 OR S26	Search modes - Boolean/Phrase	Interface - EBSCOhost Research Databases<br>Search Screen - Advanced Search<br>Database - CINAHL with Full Text	92,197
S26	iNO OR LVTT OR NMBA OR NMBAs OR pECLA OR PEEP OR prone OR AB Vt	Search modes - Boolean/Phrase	Interface - EBSCOhost Research Databases<br>Search Screen - Advanced Search<br>Database - CINAHL with Full Text	18,080
S25	ARDSnet OR ECLA OR ecls OR ecmo OR extracorporeal life support* OR extra-corporeal life support* OR extracorporeal membrane oxygenat* OR extra-corporeal membrane oxygenat* OR HFOV OR high-frequency oscillation*	Search modes - Boolean/Phrase	Interface - EBSCOhost Research Databases<br>Search Screen - Advanced Search<br>Database - CINAHL with Full Text	5,854
S24	((Nitric Oxide* and inhal*)) OR ((nitrogen monoxide* and inhal*)) OR ((nitrogen oxide* and inhal*)) OR (open W1 lung*) OR (peak W1 pressure*) OR (plateau W1 pressure*) OR (positive W1 end-expiratory pressure*) OR (protect* W2 ventilat*) OR ((recruitment W2 (manoeuv* or maneuv*))) OR (tidal W1 volume*) OR (variab* W4 pressur*) OR ((varying W4 pressur*) OR (ventilat* W6 strateg*))	Search modes - Boolean/Phrase	Interface - EBSCOhost Research Databases<br>Search Screen - Advanced Search<br>Database - CINAHL with Full Text	11,737
S23	(high frequency W3 ventil*) OR (high* W4 pressur*) OR (less* W4 pressur*) OR (low* W4 pressur*) OR (lung* W1 complianc*) OR (lung* W2 recruit*) OR (mechanic* W2 ventila*) OR (membrane* W2 oxygenat*) OR (mononitro* W1 monoxide*) OR (mono-nitro* W1 monoxide*) OR (neuromuscular W2 block*)	Search modes - Boolean/Phrase	Interface - EBSCOhost Research Databases<br>Search Screen - Advanced Search<br>Database - CINAHL with Full Text	48,389
S22	"inhal* NO" OR (alveolar W2 recruit*) OR (ART W1 strateg*) OR (artificial* W2 respirat*) OR (differen* W4 pressur*) OR (endogenous W1 nitrat* W1 vasodilat*) OR (extracorporeal W2 lung* W1 assist*) OR (extracorporeal W2 oxygenat*) OR (extra-corporeal W2 oxygenat*) OR (extracorporeal W2 support*) OR (extra-corporeal W2 support*) OR ((extracorporeal* W2 circulation) OR (extra-corporeal* W2 circulation))	Search modes - Boolean/Phrase	Interface - EBSCOhost Research Databases<br>Search Screen - Advanced Search<br>Database - CINAHL with Full Text	8,984
S21	(MH "Nitric Oxide") AND inhal*	Search modes - Boolean/Phrase	Interface - EBSCOhost Research Databases<br>Search Screen - Advanced Search<br>Database - CINAHL with Full Text	895
S20	(MH "Oxygenators, Membrane")	Search modes - Boolean/Phrase	Interface - EBSCOhost Research Databases<br>Search Screen - Advanced Search<br>Database - CINAHL with Full Text	148
S19	(MH "Extracorporeal Circulation")	Search modes - Boolean/Phrase	Interface - EBSCOhost Research Databases<br>Search Screen - Advanced Search<br>Database - CINAHL with Full Text	728

© 2019 Aoyama H et al. *JAMA Network Open.*

S18	(MH "Extracorporeal Membrane Oxygenation")	Search modes - Boolean/Phrase	Interface - EBSCOhost Research Databases<br>Search Screen - Advanced Search<br>Database - CINAHL with Full Text	3,757
S17	(MH "Neuromuscular Blocking Agents+")	Search modes - Boolean/Phrase	Interface - EBSCOhost Research Databases<br>Search Screen - Advanced Search<br>Database - CINAHL with Full Text	3,861
S16	(MH "Positive-Pressure Respiration, Intrinsic")	Search modes - Boolean/Phrase	Interface - EBSCOhost Research Databases<br>Search Screen - Advanced Search<br>Database - CINAHL with Full Text	84
S15	(MH "Ventilators, Mechanical")	Search modes - Boolean/Phrase	Interface - EBSCOhost Research Databases<br>Search Screen - Advanced Search<br>Database - CINAHL with Full Text	2,279
S14	(MH "Prone Position")	Search modes - Boolean/Phrase	Interface - EBSCOhost Research Databases<br>Search Screen - Advanced Search<br>Database - CINAHL with Full Text	1,793
S13	(MH "Respiration, Artificial")	Search modes - Boolean/Phrase	Interface - EBSCOhost Research Databases<br>Search Screen - Advanced Search<br>Database - CINAHL with Full Text	17,924
S12	(MH "Tidal Volume")	Search modes - Boolean/Phrase	Interface - EBSCOhost Research Databases<br>Search Screen - Advanced Search<br>Database - CINAHL with Full Text	2,563
S11	(MH "Lung Compliance")	Search modes - Boolean/Phrase	Interface - EBSCOhost Research Databases<br>Search Screen - Advanced Search<br>Database - CINAHL with Full Text	518
S10	(MH "Pressure")	Search modes - Boolean/Phrase	Interface - EBSCOhost Research Databases<br>Search Screen - Advanced Search<br>Database - CINAHL with Full Text	7,715
S9	S1 OR S2 OR S3 OR S4 OR S5 OR S6 OR S7 OR S8	Search modes - Boolean/Phrase	Interface - EBSCOhost Research Databases<br>Search Screen - Advanced Search<br>Database - CINAHL with Full Text	24,812
S8	(bronchopulmon* W2 insufficien*) OR (cardiopulmon* W2 insufficien*) OR (lung* W2 injur*) OR (pulmon* W2 insufficien*) OR (respirat* W2 insufficien*) OR (ventilat* W1 induced) OR TI ALI OR AB ALI	Search modes - Boolean/Phrase	Interface - EBSCOhost Research Databases<br>Search Screen - Advanced Search<br>Database - CINAHL with Full Text	7,869
S7	(acute W1 bronchopulmonary W1 fail*) OR (acute W1 bronchopulmonary W1 injur*) OR (acute W1 chest W1 syndrome*) OR (acute W1 lung* W1 fail*) OR (acute W1 lung* W1 injur*) OR (acute W1 pulmonary W1 fail*) OR (acute W1 pulmonary W1 injur*) OR (acute W1 respirat* W1 fail*) OR (acute W1 respirat* W1 injur*)	Search modes - Boolean/Phrase	Interface - EBSCOhost Research Databases<br>Search Screen - Advanced Search<br>Database - CINAHL with Full Text	5,282
S6	acute respiratory distress syndrome* OR adult respiratory distress syndrome* OR (ARDS OR ARDSS) OR shock lung OR (acute W1 chest W1 syndrome*) OR (ventilat* W1 induced)	Search modes - Boolean/Phrase	Interface - EBSCOhost Research Databases<br>Search Screen - Advanced Search<br>Database - CINAHL with Full Text	6,639
S5	(MH "Respiratory Failure")	Search modes - Boolean/Phrase	Interface - EBSCOhost Research Databases<br>Search Screen - Advanced Search<br>Database - CINAHL with Full Text	6,135
S4	(MH "Lung Injury") OR (MH "Acute Lung Injury")	Search modes - Boolean/Phrase	Interface - EBSCOhost Research Databases<br>Search Screen - Advanced Search<br>Database - CINAHL with Full Text	2,768
S3	(MH "Ventilator-Induced Lung Injury+")	Search modes - Boolean/Phrase	Interface - EBSCOhost Research Databases<br>Search Screen - Advanced Search<br>Database - CINAHL with Full Text	5,252
S2	(MH "Acute Chest Syndrome")	Search modes - Boolean/Phrase	Interface - EBSCOhost Research Databases<br>Search Screen - Advanced Search<br>Database - CINAHL with Full Text	83
S1	(MH "Respiratory Distress Syndrome, Acute")	Search modes - Boolean/Phrase	Interface - EBSCOhost Research Databases<br>Search Screen - Advanced Search<br>Database - CINAHL with Full Text	6,025

© 2019 Aoyama H et al. *JAMA Network Open.*

eAppendix 3. Assessment of Heterogeneity, Consistency, and Intransitivity

Clinical heterogeneity (i.e., between-trials differences in patients, treatments, outcomes characteristics etc) across eligible studies was assessed by examining details of participants and baseline characteristics. Statistical heterogeneity in individual pair-wise meta-analyses was explored with univariate tests for heterogeneity such as the estimates of the I^2 statistic. Statistical heterogeneity was considered moderate when I^2=50-74% and high when $I^2 \geq$75%.[1]

Consistency (i.e., between-trials differences in the underlying treatment effects between comparisons) was assessed using the node splitting method when closed loops exist within the network.[2] Transitivity (the assumption that all treatments are equally likely candidates for the patients in the network and therefore any indirect comparisons are valid) is also important assumption of network meta-analysis. Intransitivity was assessed by determining the distributions of potential effect modifiers between comparisons with the potential impact of these distributions being assessed in subgroup analyses.[2]

© 2019 Aoyama H et al. *JAMA Network Open.*

eAppendix 4. Network Metaregression With Treatment-by-Covariate Interactions to Measure the Impact of Age and ARDS Severity

To adjust for differences in study-level characteristics, we conducted network meta-regression with treatment-by-covariate interactions to measure the impact of age and ARDS severity (i.e., PaO_2/FIO_2 ratio) on the estimated treatment effect. Because there was no impact of age or ARDS severity on the estimated treatment effect, we did not adjust these covariates in the final analyses (eFigure 1).

© 2019 Aoyama H et al. *JAMA Network Open.*

eFigure 1. Results of Network Metaregression for Age (a) and ARDS Severity (b)

(a) Treatment effect of each intervention versus covariate (Age): Lung Protective Ventilation (Reference)

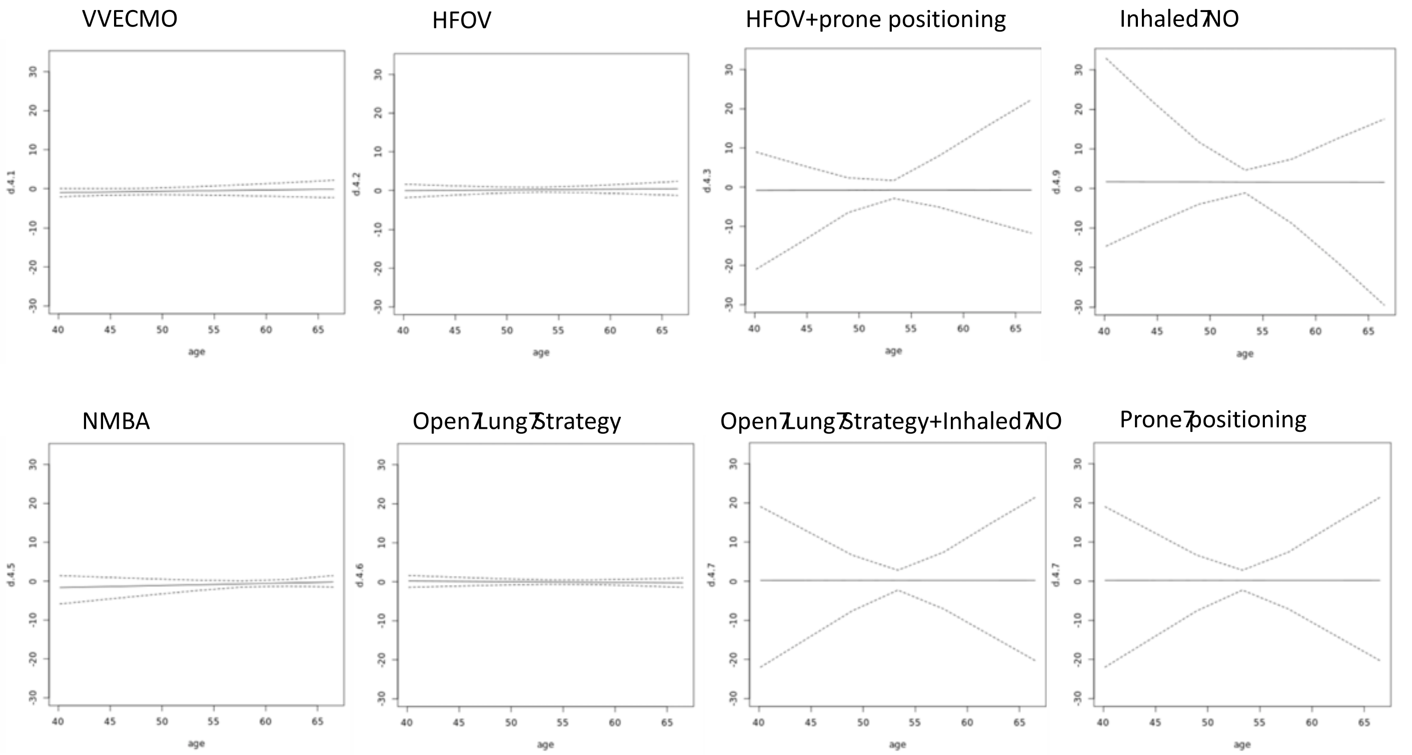

(b) Treatment effect of each intervention versus covariate (ARDS severity - PaO_2/FIO_2 ratio): Lung Protective Ventilation (Reference)

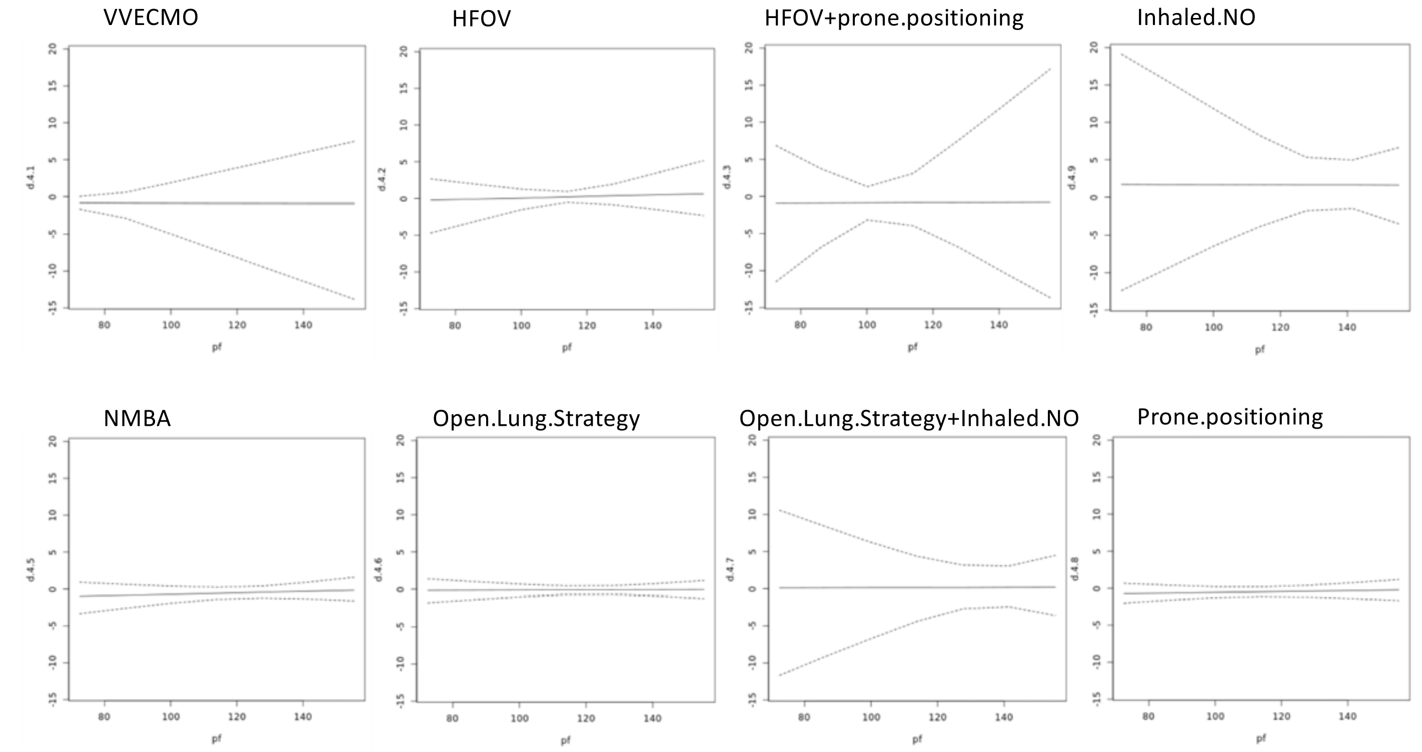

HFOV: high frequency oscillatory ventilation, inhaled NO: inhaled nitric oxide, NMBA: neuromuscular blockade, Open lung strategy: open lung strategy using recruitment maneuver and/or higher positive end-expiratory pressure, VV ECMO: venovenous extracorporeal membrane oxygenation

© 2019 Aoyama H et al. *JAMA Network Open.*

eFigure 2. Forest Plots of Direct, Indirect, and Pooled Comparisons for Primary Outcome

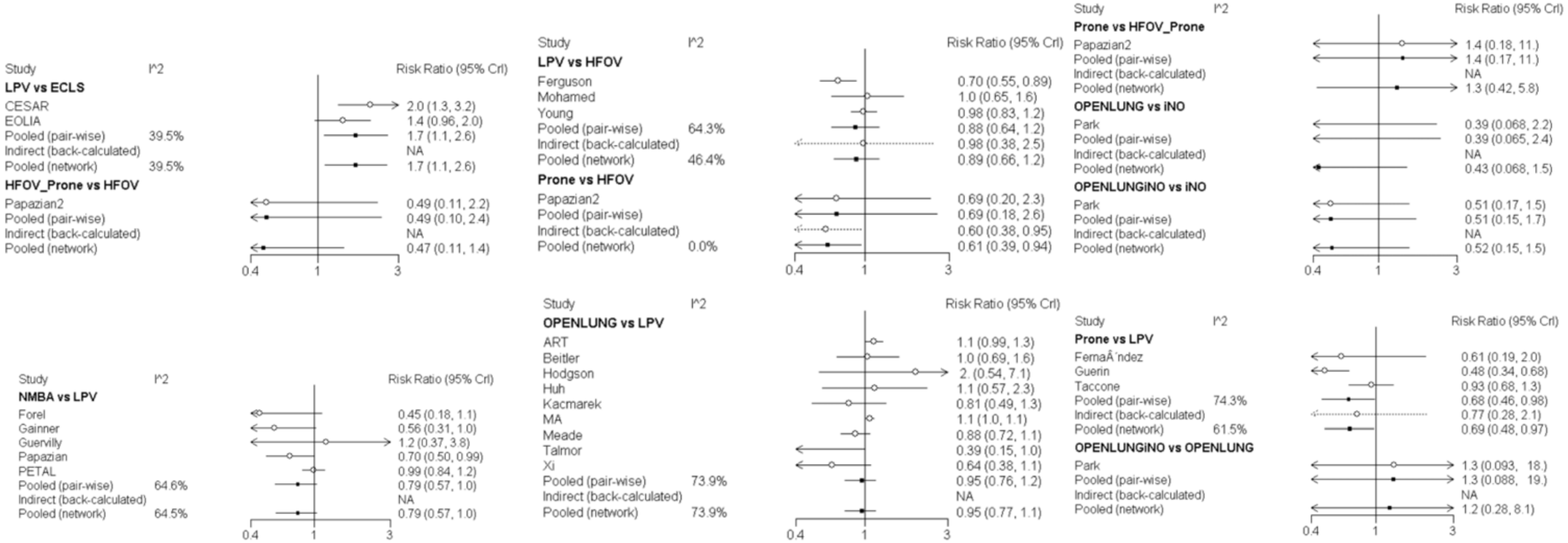

ECLS means VV ECMO here : venovenous extracorporeal membrane oxygenation, HFOV: high frequency oscillatory ventilation, iNO: inhaled nitric oxide, LPV: lung protective ventilation, NMBA: neuromuscular blockade, OPENLUNG: open lung strategy using recruitment maneuver and/or higher positive end-expiratory pressure

© 2019 Aoyama H et al. *JAMA Network Open.*

eFigure 3. Forest Plots of Direct, Indirect, and Pooled Comparisons for Secondary Outcome

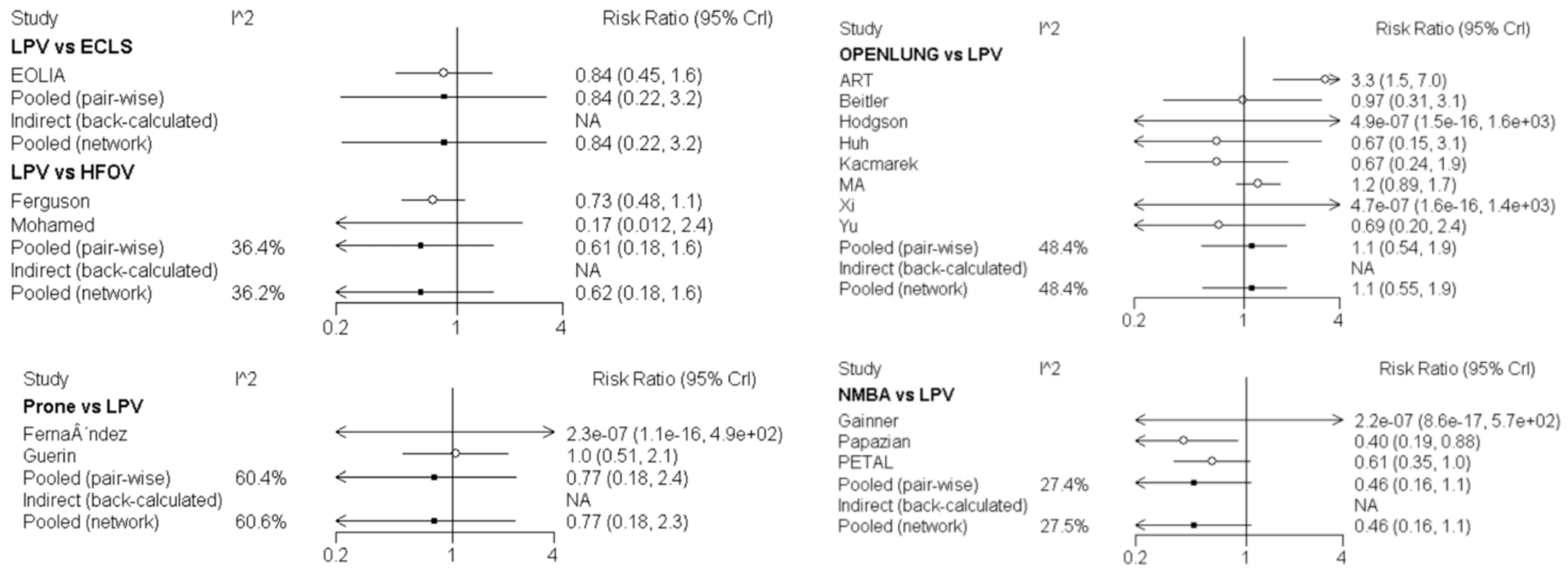

ECLS means VV ECMO here : venovenous extracorporeal membrane oxygenation, HFOV: high frequency oscillatory ventilation, iNO: inhaled nitric oxide, LPV: lung protective ventilation, NMBA: neuromuscular blockade, OPENLUNG: open lung strategy using recruitment maneuver and/or higher positive end-expiratory pressure

© 2019 Aoyama H et al. *JAMA Network Open.*

eFigure 4. Risk-of-Bias Graph for All Eligible Studies

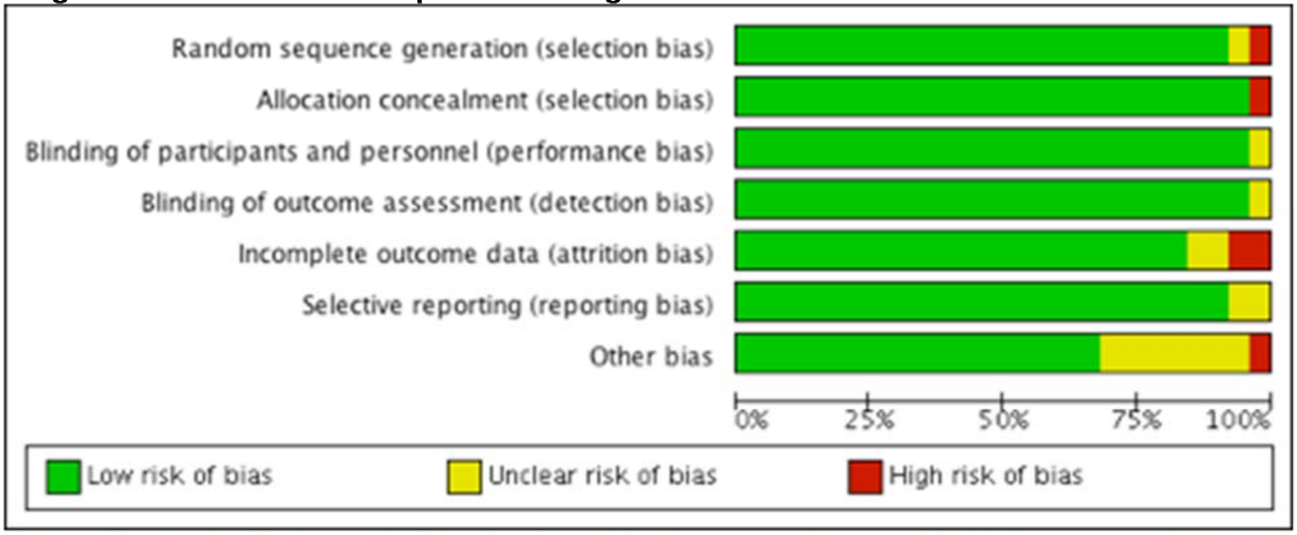

Risk of bias graph: review authors' judgements about each risk of bias item presented as percentages across all included studies.

© 2019 Aoyama H et al. *JAMA Network Open.*

eFigure 5. Risk-of-Bias Summary for All Eligible Studies

	Random sequence generation (selection bias)	Allocation concealment (selection bias)	Blinding of participants and personnel (performance bias)	Blinding of outcome assessment (detection bias)	Incomplete outcome data (attrition bias)	Selective reporting (reporting bias)	Other bias
Beitler 2018	+	+	+	+	+	+	+
Brower 2004	+	+	+	+	−	+	?
Combes 2018	+	+	+	+	+	+	?
Ferguson 2013	+	+	+	+	+	+	?
Ferna´ ndez 2008	+	+	+	+	+	+	?
Forel 2006	+	+	+	+	+	+	+
Gainnier 2004	+	+	+	+	+	+	+
Guerin 2013	+	+	+	+	+	+	+
Guervilly 2017	+	+	+	+	+	+	+
Hodgson 2011	+	+	+	+	+	+	+
Huh 2009	+	+	+	+	?	+	+
Kacmarek 2016	+	+	+	+	+	+	+
Meade 2008	+	+	+	+	+	+	+
Mercat 2008	+	+	+	+	+	+	+
Mohamed 2016	+	+	+	+	−	+	+
Papazian 2005	?	+	+	+	+	?	+
Papazian 2010	+	+	+	+	+	+	+
Park 2003	−	−	+	+	+	?	?
Peek 2009	+	+	+	+	+	+	?
PETAL 2019	+	+	+	+	+	+	+
Taccone 2009	+	+	+	+	+	+	?
Talmor 2008	+	+	+	+	+	+	+
Writing Group for ART 2017	+	+	+	+	+	+	+
Xi 2010	+	+	?	?	?	+	−
Young 2013	+	+	+	+	+	+	+

Risk of bias summary: review authors' judgements about each risk of bias item for each included study.

© 2019 Aoyama H et al. *JAMA Network Open.*

eFigure 6. Funnel Plots for Primary (a) and (b) Secondary Outcomes

(a) primary outcome

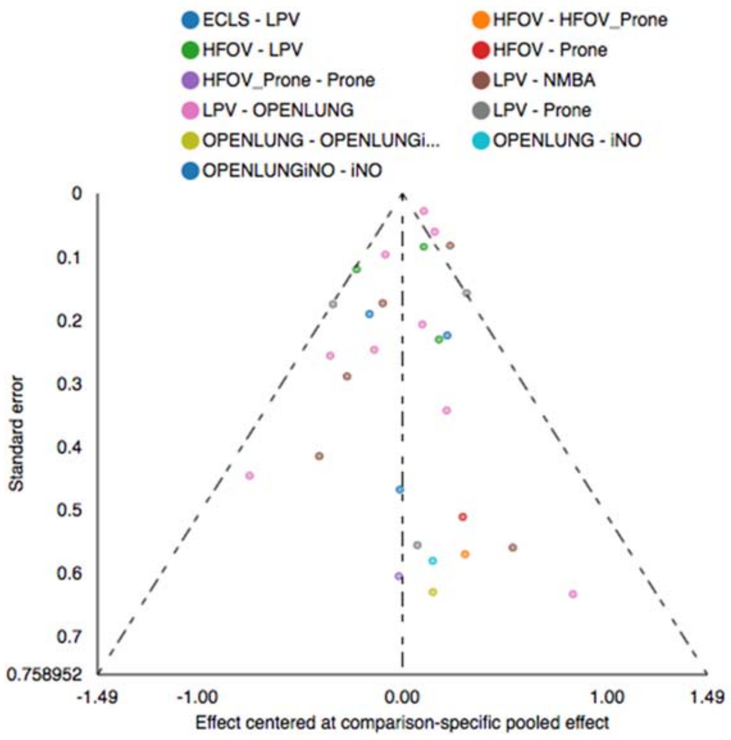

(b) secondary outcome

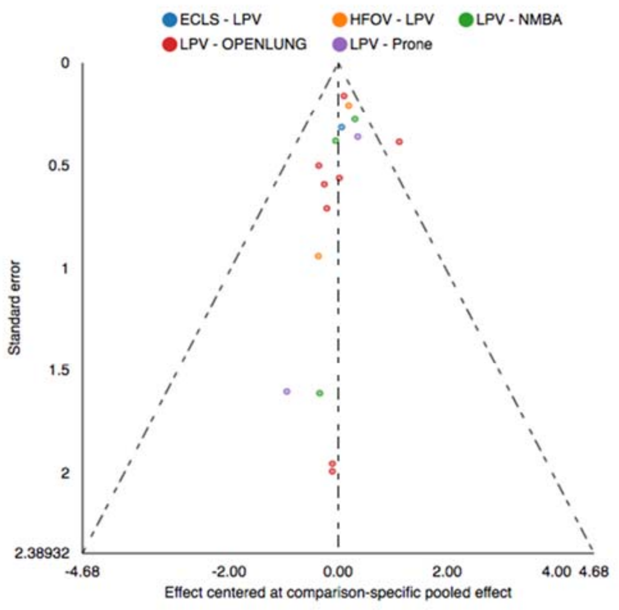

ECLS means VV ECMO here : venovenous extracorporeal membrane oxygenation, HFOV: high frequency oscillatory ventilation, iNO: inhaled nitric oxide, LPV: lung protective ventilation, NMBA: neuromuscular blockade, OPENLUNG: open lung strategy using recruitment maneuver and/or higher positive end-expiratory pressure

© 2019 Aoyama H et al. *JAMA Network Open.*

eFigure 7. Gelman Plots for Model Convergence With 4 Chains and 2 000 000 Iterated Simulations, Discarding the Initial 1 500 000 Iterations as Burn-in on Primary (a) and (b) Secondary Outcomes

(a) primary outcome

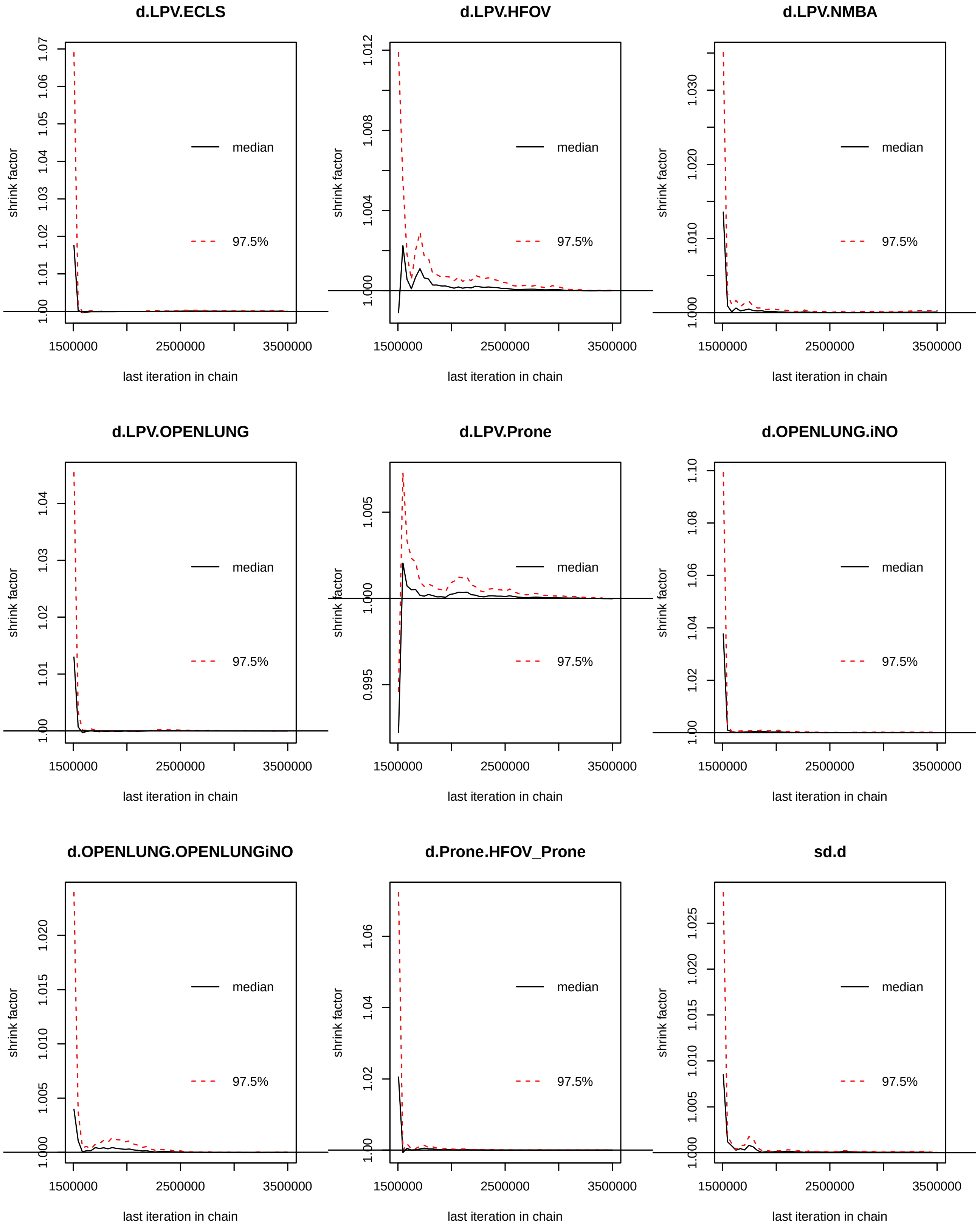

© 2019 Aoyama H et al. *JAMA Network Open.*

(b) secondary outcome

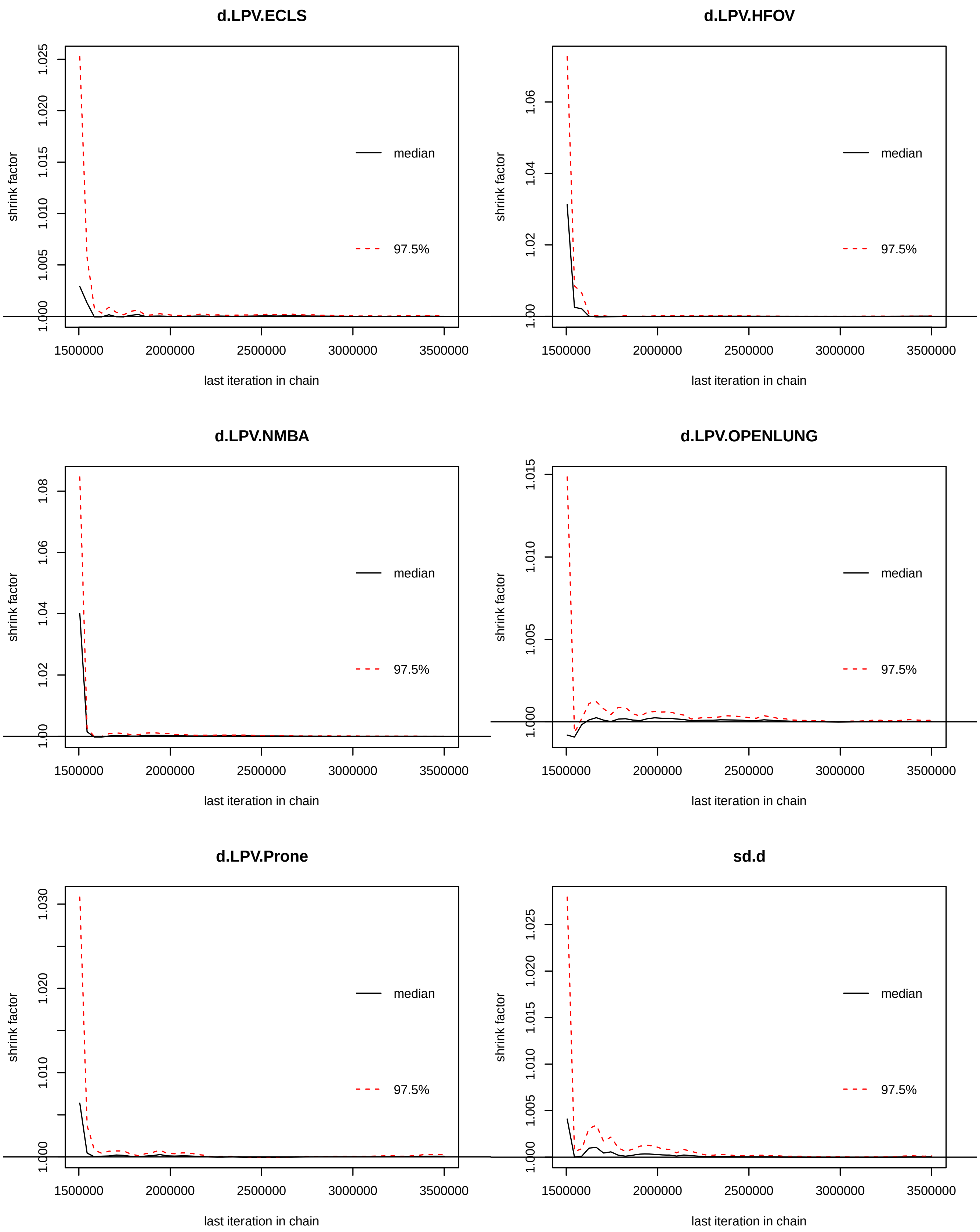

© 2019 Aoyama H et al. *JAMA Network Open.*

ECLS means VV ECMO here : venovenous extracorporeal membrane oxygenation, HFOV: high frequency oscillatory ventilation, iNO: inhaled nitric oxide, LPV: lung protective ventilation, NMBA: neuromuscular blockade, OPENLUNG: open lung strategy using recruitment maneuver and/or higher positive end-expiratory pressure

© 2019 Aoyama H et al. *JAMA Network Open.*

eFigure 8. Ranking Probabilities for the Effect of Interventions on the Outcomes

(a) Ranking Probabilities for the Effect of Interventions on 28-day mortality

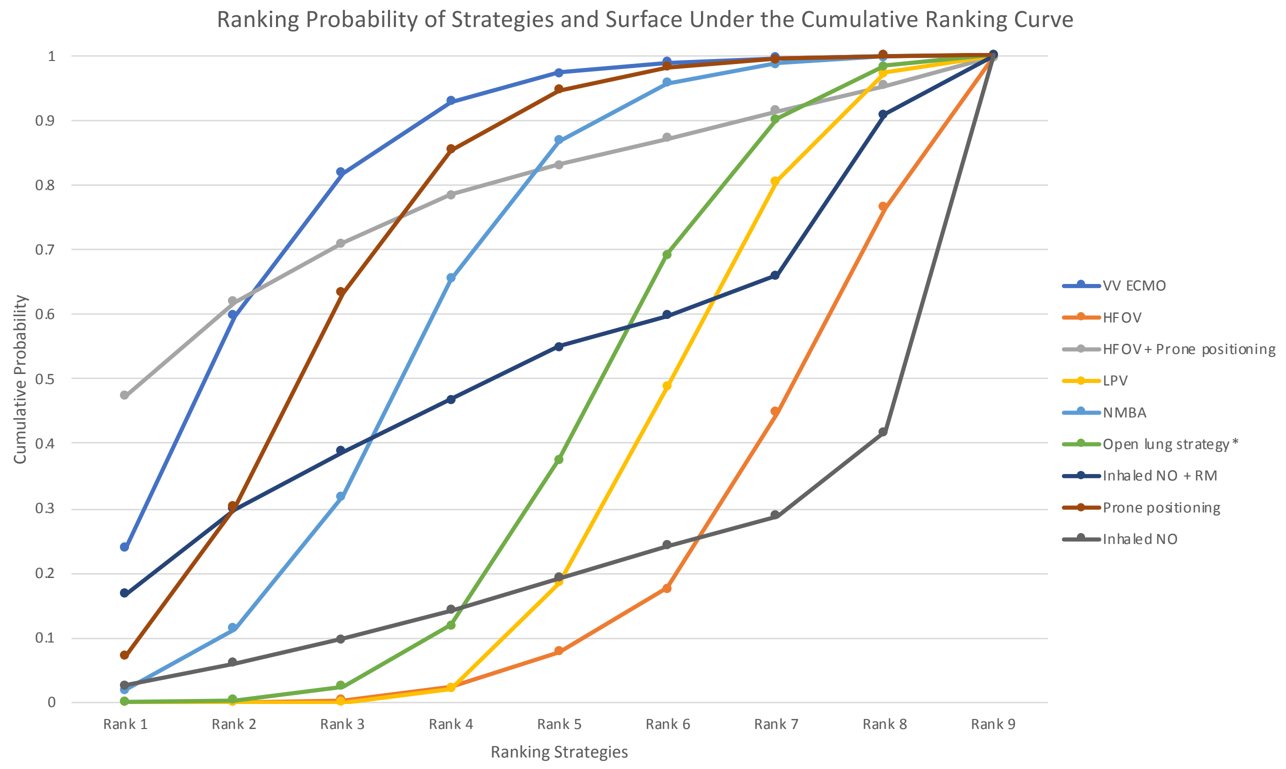

	Surface Under the Cumulatve Ranking
VV ECMO	0.82
HFOV	0.19
HFOV+ Prone positioning	0.77
LPV	0.31
NMBA	0.62
Open lung strategy*	0.39
Inhaled NO + RM	0.51
Prone positioning	0.72
Inhaled NO	0.18

(b) Ranking Probabilities for the Effect of Interventions on Barotrauma

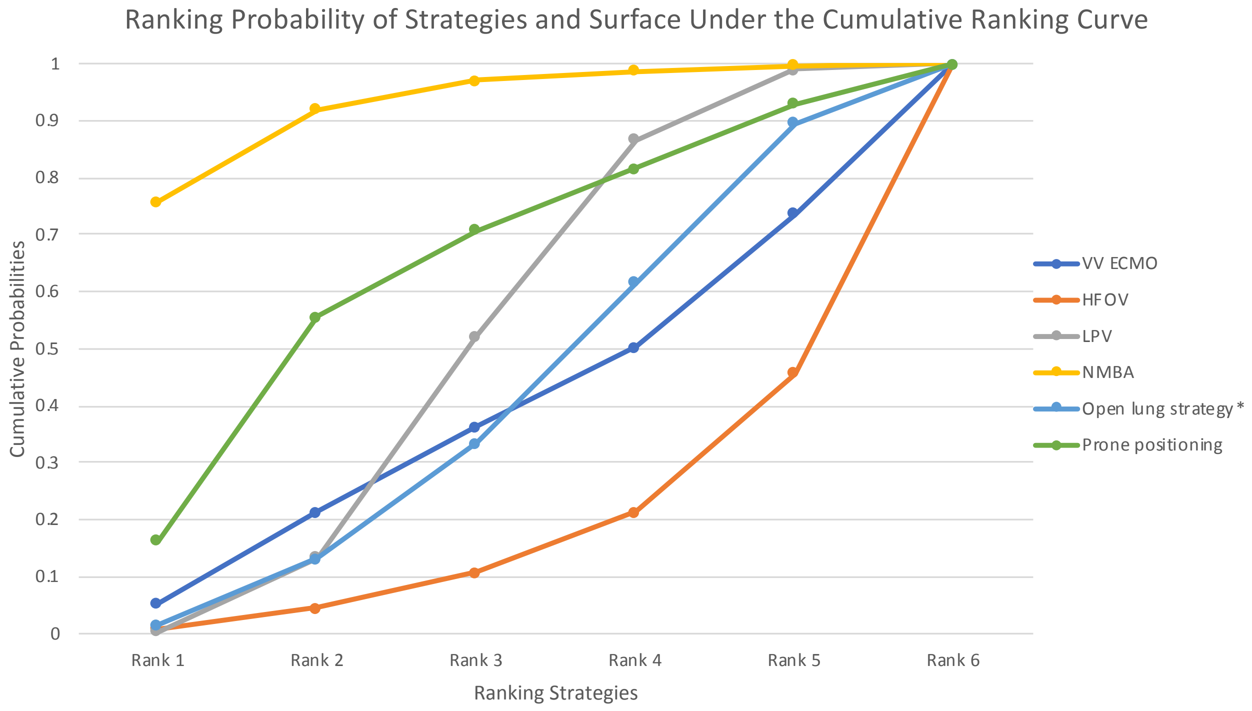

	Surface Under the Cumulatve Ranking Curve
VV ECMO	0.37
HFOV	0.17
LPV	0.50
NMBA	0.93
Open lung strategy*	0.40
Prone positioning	0.63

Each line indicates an intervention. The surface area under the curve is constructed on the basis of the ranking probabilities for all interventions. The x-axis represents the ranking of strategies where the best through worst interventions are ranked in numerical order. The y-axis represents the probability of each ranking.

© 2019 Aoyama H et al. *JAMA Network Open.*

eTable 1. Detailed Explanation for Excluded Studies While Full-Text Assessment

Authors	Published year	Reason for exclusion
ARDS Network	2000	Irrelevant intervention (low v.s. high tidal volume)
Bein et al	2013	Irrelevant intervention (extracorporeal CO_2 removal)
Bollen et al	2005	Low tidal volume ventilation was not applied (VT=8.4ml/kg in control)
Cavalcanti et al	2017	Duplication
Crucean et al	2010	French report of the Peek 2009 results
Dellinger et al	1998	No information on tidal volume limitation
Derdak et al	2002	Low tidal volume ventilation was not applied (VT 6-10)
Gattinoni et al	2001	Low tidal volume ventilation was not applied (VT over 10)
Gerlach et al	2003	Low tidal volume ventilation was not applied (VT over 10)
Johannigman et al	1997	Irrelevant intervention
Kung et al	2019	Low tidal volume ventilation was not applied (VT over 8)
Lall et al	2015	Duplication of OSCAR study
Mancebo et al	2006	Low tidal volume ventilation was not applied (VT was set at 10)
Mehta et al	2001	Mortality was not reported<br>Low tidal volume ventilation was not applied
Michael et al	1998	Low tidal volume ventilation was not applied
Needham et al	2011	Commentary
Needham et al	2012	Commentary
Rialp et al	2001	Low tidal volume ventilation was not applied
Taylor et al	2004	No information on tidal volume limitation
Troncy et al	1998	Low tidal volume ventilation was not applied (VT was set at 10)
Yi et al	2005	Mortality was not reported
Chan et al	2007	Irrelevant study design
Yu et al	2017	All ARDS severity was included: irrelevant population (unable to extract the data of moderate to severe)
Voggenreiter	2005	All ARDS severity was included: irrelevant population (unable to extract the data of moderate to severe)

© 2019 Aoyama H et al. *JAMA Network Open.*

eTable 2. Comparison of the Included Interventions in Risk Ratio (95% CrI) for 28-Day Mortality

**VV ECMO**	1.88 (1.12, 3.24)	0.87 (0.19, 2.90)	1.67 (1.10, 2.58)	1.30 (0.77, 2.14)	1.59 (0.99, 2.56)	1.43 (0.34, 6.60)	1.15 (0.66, 2.01)	2.48 (0.67, 10.9)
	**HFOV**	0.47 (0.11, 1.44)	0.89 (0.66, 1.20)	0.70 (0.48, 1.02)	0.85 (0.58, 1.19)	0.76 (0.18, 3.50)	0.61 (0.39, 0.95)	1.32 (0.35, 5.51)
		**HFOV + Prone positioning**	1.89 (0.61, 8.49)	1.48 (0.45, 6.63)	1.80 (0.56, 8.01)	1.67 (0.26, 13.36)	1.31 (0.41, 5.83)	2.93 (0.49, 22.0)
			**LPV**	0.79 (0.57, 1.02)	0.96 (0.77, 1.14)	0.86 (0.22, 3.83)	0.69 (0.48, 0.98)	1.48 (0.42, 6.08)
				**NMBA**	1.22 (0.90, 1.72)	1.10 (0.28, 5.04)	0.88 (0.56, 1.40)	1.91 (0.51, 8.23)
					**Open lung strategy using RM and/or higher PEEP**	0.90 (0.24, 3.91)	0.72 (0.48, 1.11)	1.55 (0.44, 6.28)
						**Inhaled NO + RM**	0.80 (0.18, 3.22)	1.74 (0.51, 5.94)
							**Prone positioning**	2.15 (0.59, 8.99)
								**Inhaled NO**

Each cell gives the effect of the column-defining intervention relative to the row-defining intervention.

HFOV: high frequency oscillatory ventilation, inhaled NO: inhaled nitric oxide, LPV: lung protective ventilation, NMBA: neuromuscular blockade, PEEP: positive end-expiratory pressure, RM: recruitment maneuver, VV ECMO: venovenous extracorporeal membrane oxygenation

© 2019 Aoyama H et al. *JAMA Network Open.*

eTable 3. Network Meta-analysis for 28-Day Mortality and Quality of Evidence Assessment

Source	Risk of Bias	Heterogenity	Indirectness	Impresicion	Others	Direct Risk Ratio (95%Crl)	QOE	Indirect Risk Ratio (95%Crl)	QOE	Node splitting	Network Risk Ratio (95%Crl)	QOE
**LPV (reference)**				OIS=144								
ECLS	Not serious	Serious[b]	Not serious	Not serious	No	0.59 (0.38, 0.91)	Moderate	NA[f]	NA[f]	NA[g]	0.60 (0.38, 0.93)	Moderate
HFOV	Not serious	Serious[b]	Not serious	Serious[d]	No	1.14 (0.83, 1.59)	Low	1.02 (0.28, 3.70)	Very low	p=0.823	1.12 (0.83, 1.54)	Low
HFOV_Prone	Not serious	Serious[b]	Serious	Not serious	No	NA[f]	NA[f]	0.53 (0.12, 1.60)	Very low	NA[g]	0.53 (0.12, 1.60)	Very low
NMBA	Not serious	Not serious	Not serious	Not serious	No	0.79 (0.57, 1.00)	High	NA[f]	NA[f]	NA[g]	0.79 (0.57, 1.02)	High
OPENLUNG	Not serious	Serious[b]	Not serious	Serious[d]	No	0.95 (0.77, 1.10)	Low	NA[f]	NA[f]	NA[g]	0.96 (0.77, 1.14)	Low
OPENLUNGiNO	Serious[a]	Serious[b]	Serious	Not serious	No	NA[f]	NA[f]	0.86 (0.22, 3.83)	Very low	NA[g]	0.86 (0.22, 3.83)	Very low
Prone	Not serious	Serious[b]	Not serious	Serious[d]	No	0.68 (0.46, 098)	Low	0.77 (0.28, 2.10)	Very low	p=0.806	0.69 (0.48, 0.98)	Low
iNO	Serious[a]	Serious[b]	Serious	Not serious	No	NA[f]	NA[f]	1.48 (0.42,6.08)	Very low	NA[g]	1.48 (0.42,6.08)	Very low
**ECLS (reference)**				OIS=200								
HFOV	Not serious	Serious[b]	Serious	Serious[c]	No	NA[f]	NA[f]	1.88 (1.12, 3.24)	Low	NA[g]	1.88 (1.12, 3.24)	Low
HFOV_Prone	Not serious	Serious[b]	Serious	Serious[c]	No	NA[f]	NA[f]	0.87 (0.19, 2.90)	Very low	NA[g]	0.87 (0.19, 2.90)	Very low
NMBA	Not serious	Serious[b]	Serious	Serious[c]	No	NA[f]	NA[f]	1.30 (0.77, 2.14)	Very low	NA[g]	1.30 (0.77, 2.14)	Very low
OPENLUNG	Not serious	Serious[b]	Serious	Serious[c]	No	NA[f]	NA[f]	1.59 (0.99, 2.56)	Very low	NA[g]	1.59 (0.99, 2.56)	Very low
OPENLUNGiNO	Serious[a]	Serious[b]	Serious	Serious[c]	No	NA[f]	NA[f]	1.43 (0.34, 6.60)	Very low	NA[g]	1.43 (0.34, 6.60)	Very low
Prone	Not serious	Serious[b]	Serious	Serious[c]	No	NA[f]	NA[f]	1.15 (0.66, 2.01)	Very low	NA[g]	1.15 (0.66, 2.01)	Very low
iNO	Serious[a]	Serious[b]	Serious	Serious[c]	No	NA[f]	NA[f]	2.48 (0.67, 10.9)	Very low	NA[g]	2.48 (0.67, 10.9)	Very low
**HFOV (reference)**				OIS=161								
HFOV_Prone	Not serious	Not serious	Not serious	Very serious[e]	No	0.49 (0.10, 2.40)	Low	NA[f]	NA[f]	NA[g]	0.47 (0.11, 1.44)	Low
NMBA	Not serious	Serious[b]	Serious	Not serious	No	NA[f]	NA[f]	0.70 (0.48, 1.02)	Moderate	NA[g]	0.70 (0.48, 1.02)	Moderate
OPENLUNG	Not serious	Serious[b]	Serious	Not serious	No	NA[f]	NA[f]	0.85 (0.58, 1.19)	Low	NA[g]	0.85 (0.58, 1.19)	Low
OPENLUNGiNO	Serious[a]	Serious[b]	Serious	Not serious	No	NA[f]	NA[f]	0.76 (0.18, 3.50)	Very low	NA[g]	0.76 (0.18, 3.50)	Very low
Prone	Not serious	Not serious	Not serious	Very serious[e]	No	0.69 (0.18, 2.60)	Low	0.60 (0.38, 0.95)	Moderate	p=0.798	0.61 (0.39, 0.95)	Moderate
iNO	Serious[a]	Serious[b]	Serious	Not serious	No	NA[f]	NA[f]	1.32 (0.35, 5.51)	Very low	NA[g]	1.32 (0.35, 5.51)	Very low
**HFOV_Prone (reference)**				OIS=202								
NMBA	Not serious	Serious[b]	Serious	Serious[c]	No	NA[f]	NA[f]	1.48 (0.45, 6.63)	Very low	NA[g]	1.48 (0.45, 6.63)	Very low
OPENLUNG	Not serious	Serious[b]	Serious	Serious[c]	No	NA[f]	NA[f]	1.80 (0.56, 8.01)	Very low	NA[g]	1.80 (0.56, 8.01)	Very low
OPENLUNGiNO	Serious[a]	Serious[b]	Serious	Serious[c]	No	NA[f]	NA[f]	1.67 (0.26, 13.4)	Very low	NA[g]	1.67 (0.26, 13.4)	Very low
Prone	Not serious	Not serious	Not serious	Very serious[e]	No	1.40 (0.17, 11.0)	Low	NA[f]	NA[f]	NA[g]	1.31 (0.41, 5.83)	Low
iNO	Serious[a]	Serious[b]	Serious	Serious[c]	No	NA[f]	NA[f]	2.93 (0.49, 22.0)	Very low	NA[g]	2.93 (0.49, 22.0)	Very low
**NMBA (reference)**				OIS=183								
OPENLUNG	Not serious	Serious[b]	Serious	Serious[c]	No	NA[f]	NA[f]	1.50 (0.90, 2.30)	Very low	NA[g]	1.22 (0.90, 1.72)	Very low
OPENLUNGiNO	Serious[a]	Serious[b]	Serious	Serious[c]	No	NA[f]	NA[f]	1.80 (0.37, 13.0)	Very low	NA[g]	1.10 (0.28, 5.04)	Very low
Prone	Not serious	Serious[b]	Serious	Serious[c]	No	NA[f]	NA[f]	1.00 (0.62, 1.80)	Very low	NA[g]	0.88 (0.56, 1.40)	Very low
iNO	Serious[a]	Serious[b]	Serious	Serious[c]	No	NA[f]	NA[f]	3.40 (0.89, 23.0)	Very low	NA[g]	1.91 (0.51, 8.23)	Very low
**OPENLUNG (reference)**				OIS=122								
OPENLUNGiNO	Serious[a]	Not serious	Not serious	Very serious[e]	No	1.30 (0.09, 19.0)	Very low	NA[f]	NA[f]	NA[g]	0.90 (0.24, 3.91)	Very low
Prone	Not serious	Serious[b]	Serious	Not serious	No	NA[f]	NA[f]	0.72 (0.48, 1.11)	Very low	NA[g]	0.72 (0.48, 1.11)	Very low
iNO	Serious[a]	Not serious	Not serious	Very serious[e]	No	2.56 (0.42, 15.38)	Very low	NA[f]	NA[f]	NA[g]	1.55 (0.44, 6.28)	Very low
**OPENLUNGiNO (reference)**				OIS=179								
Prone	Serious[a]	Serious[b]	Serious	Serious[c]	No	NA[f]	NA[f]	0.80 (0.18, 3.22)	Very low	NA[g]	0.80 (0.18, 3.22)	Very low
iNO	Serious[a]	Not serious	Not serious	Very serious[e]	No	1.96 (0.59, 6.67)	Very low	NA[f]	NA[f]	NA[g]	1.74 (0.51, 5.94)	Very low
**Prone (rference)**				OIS=199								
iNO	Serious[a]	Serious[b]	Serious	Serious[c]	No	NA[f]	NA[f]	2.15 (0.59, 8.99)	Very low	NA[g]	2.15 (0.59, 8.99)	Very low

When there was no direct comparison, the quality of evidence of indirect comparisons was inferred from the lower quality of evidence among direct comparisons within the first-order loop.

a: The sensitivity analysis excluding studies with high risk of bias greatly changed the RR estimate

b: Moderate heterogeneity (I-squared = 30-60%) or substantial heterogeneity (I-squared = 60-90%) was found

c: The sample size did not reach the optimal information size (OIS)

d: The OIS met, but CrI included RR of 1.0

e: 95%CrI included benefit (RR=0.8) and harm (RR=1.25)

f: Not available due to no direct comparison or no indirect comparison

g: Not available, due to eithr no direct comaprison or no indirect estimate secaondary to lack of first closed loop

© 2019 Aoyama H et al. *JAMA Network Open.*

eTable 4. Results of Sensitivity Analysis for 28-Day Mortality

Source	Number of patients	Number of trials	Network Risk Ratio (95%Crl)
**LPV (reference)**			
VV ECMO	429	2	0.60 (0.39, 0.93)
HFOV	1343	2	1.20 (0.81, 1.70)
HFOV and Prone positioning		Indirect evidence	0.55 (0.12, 1.70)
NMBA	956	5	0.79 (0.57, 1.00)
Open lung strategy [a]	3338	7	1.00 (0.77, 1.30)
Prone positioning	848	3	0.69 (0.47, 0.99)
**VV ECMO (reference)**			
HFOV		Indirect evidence	1.90 (1.10, 3.40)
NMBA		Indirect evidence	1.70 (1.00, 2.70)
HFOV and Prone positioning		Indirect evidence	0.91 (0.20, 3.10)
Open lung strategy [a]		Indirect evidence	1.70 (1.00, 2.70)
Prone positioning		Indirect evidence	1.10 (0.65, 2.10)
**HFOV (reference)**			
HFOV and Prone positioning	26	1	0.47 (0.11, 1.40)
NMBA		Indirect evidence	0.68 (0.41, 1.00)
Open lung strategy [a]		Indirect evidence	0.87 (0.55, 1.30)
Prone positioning	26	1	0.59 (0.36, 0.95)
**HFOV and Prone positioning (reference)**			
NMBA		Indirect evidence	1.40 (0.44, 6.50)
Open lung strategy [a]		Indirect evidence	1.80 (0.57, 8.20)
Prone positioning	26	1	1.30 (0.40, 5.40)
**NMBA (reference)**			
Open lung strategy [a]		Indirect evidence	1.30 (0.88, 1.90)
Prone positioning		Indirect evidence	0.87 (0.55, 1.40)
**Open lung strategy[a] (reference)**			
Prone positioning		Indirect evidence	0.68 (0.43, 1.10)

a: Open lung strategy: open lung strategy with RM and/or higher PEEP

CrI: credible intervals , HFOV: high frequency oscillatory ventilation, inhaled NO: inhaled nitric oxide, LPV: lung protective ventilation, NMBA: neuromuscular blockade, PEEP: positive end-expiratory pressure, RM: recruitment maneuver, VV ECMO: venovenous extracorporeal membrane oxygenation

© 2019 Aoyama H et al. *JAMA Network Open.*

eTable 5. Results of Preliminary Analysis Excluding Studies Without Description of Cointerventions

Source	Number of patients	Number of trials	Network Risk Ratio (95%CrI)
**LPV (reference)**			
VV ECMO	429	2	0.60 (0.37, 0.95)
HFOV	548	1	1.20 (0.79, 1.70)
NMBA	932	4	0.55 (0.13, 1.80)
Open lung strategy[a]	3077	5	0.98 (0.71, 1.30)
Prone positioning	808	2	0.69 (0.46, 0.99)
**VV ECMO (reference)**			
HFOV		Indirect evidence	2.00 (1.10, 3.60)
NMBA		Indirect evidence	1.30 (0.70, 2.20)
Open lung strategy[a]		Indirect evidence	1.60 (0.93, 2.80)
Prone positioning		Indirect evidence	1.20 (0.63, 2.10)
**HFOV (reference)**			
NMBA		Indirect evidence	0.66 (0.39, 1.10)
Open lung strategy[a]		Indirect evidence	0.84 (0.51, 1.40)
Prone positioning		Indirect evidence	0.59 (0.36, 1.00)
**NMBA (reference)**			
Open lung strategy[a]		Indirect evidence	1.30 (0.82, 2.00)
Prone positioning		Indirect evidence	0.89 (0.55, 1.50)
**Open lung strategy[a] (reference)**			
Prone positioning		Indirect evidence	0.70 (0.43, 1.20)

a: Open lung strategy: open lung strategy with RM and/or higher PEEP
CrI: credible intervals , HFOV: high frequency oscillatory ventilation, LPV: lung protective ventilation, NMBA: neuromuscular blockade, PEEP: positive end-expiratory pressure, VV ECMO: venovenous extracorporeal membrane oxygenation

© 2019 Aoyama H et al. *JAMA Network Open.*

eTable 6. Results of Analysis Using Poisson Models for the Primary Outcome Adjusting Different Timing of the Measurement

Source	Number of patients	Number of trials	Network Hazard Ratio (95%Crl)
**LPV (reference)**			
VV ECMO	429	2	0.58 (0.39, 0.88)
HFOV	1403	3	1.10 (0.86, 1.50)
HFOV and Prone positioning		Indirect evidence	0.56 (0.11, 2.10)
NMBA	956	5	0.87 (0.63, 1.10)
Open lung strategy[a]	3452	10	1.00 (0.80, 1.20)
Inhaled NO + RM		Indirect evidence	2.20 (0.37, 18.0)
Prone positioning	848	3	0.70 (0.50, 0.98)
Inhaled NO		Indirect evidence	2.20 (0.41,18.0)
**VV ECMO (reference)**			
HFOV		Indirect evidence	1.90 (1.20, 3.20)
HFOV + Prone positioning		Indirect evidence	0.95 (0.18, 3.90)
NMBA		Indirect evidence	1.50 (0.88, 2.40)
Open lung strategy[a]		Indirect evidence	1.70 (1.1, 2.70)
Inhaled NO and RM		Indirect evidence	3.80 (0.60, 33.0)
Prone positioning		Indirect evidence	1.20 (0.72, 2.00)
Inhaled NO		Indirect evidence	3.80 (0.66, 33.0)
**HFOV (reference)**			
HFOV and Prone positioning	26	1	0.49 (0.10, 1.80)
NMBA		Indirect evidence	0.77 (0.50, 1.10)
Open lung strategy[a]		Indirect evidence	0.90 (0.61, 1.20)
Inhaled NO and RM		Indirect evidence	2.00 (0.32, 16.0)
Prone positioning	26	1	0.62 (0.41, 0.94)
Inhaled NO		Indirect evidence	2.00 (0.35, 16.0)
**HFOV + Prone Positioning (reference)**			
NMBA		Indirect evidence	1.60 (0.39, 8.10)
Open lung strategy[a]		Indirect evidence	1.80 (0.47, 9.50)
Inhaled NO and RM		Indirect evidence	4.10 (0.42, 56.0)
Prone positioning	26	1	1.30 (0.33, 6.60)

© 2019 Aoyama H et al. *JAMA Network Open.*

Inhaled NO		Indirect evidence	4.10 (0.43, 59.0)
**NMBA (reference)**			
Open lung strategy[a]		Indirect evidence	1.20 (0.83, 1.60)
Inhaled NO and RM		Indirect evidence	2.60 (0.42, 22.0)
Prone positioning		Indirect evidence	0.80 (0.53, 1.30)
Inhaled NO		Indirect evidence	2.50 (0.47, 21.0)
**Open lung strategy[a] (reference)**			
Inhaled NO + RM	17	1	2.20 (0.42, 18.0)
Prone positioning		Indirect evidence	0.69 (0.48, 1.10)
Inhaled NO	12	1	2.20 (0.42, 18.0)
**Inhaled NO and RM (reference)**			
Prone positioning		Indirect evidence	0.31 (0.04, 2.00)
Inhaled NO	17	1	1.00 (0.23, 4.70)
**Prone positioning (reference)**			
Inhaled NO		Indirect evidence	3.20 (0.57, 26.0)

a: Open lung strategy: open lung strategy with RM and/or higher PEEP

CrI: credible intervals , HFOV: high frequency oscillatory ventilation, inhaled NO: inhaled nitric oxide, LPV: lung protective ventilation, NMBA: neuromuscular blockade, RM: recruitment maneuver, PEEP: positive end-expiratory pressure, VV ECMO: venovenous extracorporeal membrane oxygenation

© 2019 Aoyama H et al. *JAMA Network Open.*

eTable 7. Potential Scale Reduction Factor From Brooks-Gelman-Rubin Diagnostic for Model Convergence on Primary (a) and (b) Secondary Outcomes

(a) primary outcome

Per-parameter convergence diagnostics				
			**Potential scale reduction factor (PSRF)**	
**Parameter**	**Standard deviation**	**Time-series S.E.**	**Point estimate**	**97.5% quantile**
d.4.1 (LPV, ECLS)	0.21695	0.0027692	1.001	1.0038
d.4.2 (LPV, HFOV)	0.14867	0.0019185	1.0008	1.0029
d.4.5 (LPV, NMBA)	0.14812	0.0019476	1.0001	1.0009
d.4.6 (LPV, OPENLUNG)	0.10207	0.0013302	0.99989	1.0001
d.4.8 (LPV, Prone)	0.1789	0.0022496	0.99983	1.0001
d.6.7 (OPENLUNG, OPENLUNGiNO)	0.70252	0.0099859	1	1.0005
d.6.9 (OPENLUNG, iNO)	0.66949	0.0091481	1.0004	1.0016
d.8.3 (Prone, HFOV_Prone)	0.66837	0.0087217	1.0002	1.001
sd.d (Random effects standard deviation)	0.078145	0.0010233	1.0012	1.0042

(b) secondary outcome

Per-parameter convergence diagnostics				
			**Potential scale reduction factor (PSRF)**	
**Parameter**	**Standard deviation**	**Time-series S.E.**	**Point estimate**	**97.5% quantile**
d.3.1 (LPV, ECLS)	0.66142	0.0083749	0.99984	1
d.3.2 (LPV, HFOV)	0.5312	0.0067739	0.99982	0.99998
d.3.4 (LPV, NMBA)	0.44672	0.005472	1	1.0008
d.3.5 (LPV, OPENLUNG)	0.29971	0.0039105	0.99979	0.99991
d.3.6 (LPV, Prone)	0.62643	0.0082667	1.0006	1.0022
sd.d (Random effects standard deviation)	0.27555	0.0037402	0.99974	0.99985

ECLS means VV ECMO here : venovenous extracorporeal membrane oxygenation, HFOV: high frequency oscillatory ventilation, iNO: inhaled nitric oxide, LPV: lung protective ventilation, NMBA: neuromuscular blockade, OPENLUNG: open lung strategy using recruitment maneuver and/or higher positive end-expiratory pressure

© 2019 Aoyama H et al. *JAMA Network Open.*

eTable 8. Assessment of Model Fits for Primary (a) and (b) Secondary Outcomes

(a) primary outcome

Model fit statistics	
Residual deviance (D_{res})	50.6
Leverage (p_D)	39.9
DIC	90.6
Number of data points	50

(b) secondary outcome

Model fit statistics	
Residual deviance (D_{res})	31.7
Leverage (p_D)	21.3
DIC	52.9
Number of data points	32

Model fit was assessed by residual deviance, leverage, and the deviance information criterion.[3] A key metric of model fit is residual deviance. In a well-fitting model, we can expect the residual deviance to equal the number of independent data points.

© 2019 Aoyama H et al. *JAMA Network Open.*

eTable 9. Results of Assessment on Small Sample Size Effects

Per-parameter convergence diagnostics					
			**Potential scale reduction factor (PSRF)**		
**Parameter**	**Standard deviation**	**Time-series S.E.**	**Point estimate**	**97.5% quantile**	
d.4.1 (LPV, ECLS)	0.2724	0.003208	0.99993	1.000	
d.4.2 (LPV, HFOV)	0.2372	0.002686	1.001	1.004	
d.4.3 (LPV, NMBA)	0.2509	0.002850	1.000	1.002	
d.4.5 (LPV, OPENLUNG)	0.1738	0.002111	1.001	1.005	
d.4.6 (LPV, Prone)	0.2335	0.002646	1.000	1.000	
sd.d (Random effects standard deviation)	0.1190	0.001879	1.000	1.003	

Model fit statistics	
Residual deviance (D_{res})	32.5
Leverage (p_D)	26.5
DIC	59.0
Number of data points	32

Model fit was assessed by residual deviance, leverage, and the deviance information criterion.[3] A key metric of model fit is residual deviance. In a well-fitting model, we can expect the residual deviance to equal the number of independent data points.

ECLS means VV ECMO here : venovenous extracorporeal membrane oxygenation, HFOV: high frequency oscillatory ventilation, iNO: inhaled nitric oxide, LPV: lung protective ventilation, NMBA: neuromuscular blockade, OPENLUNG: open lung strategy using recruitment maneuver and/or higher positive end-expiratory pressure

© 2019 Aoyama H et al. *JAMA Network Open.*

eTable 10. Comparison of the Included Interventions in Risk Ratio (95% CrI) for Barotrauma

**VV ECMO**	1.35 (0.21, 8.47)	0.83 (0.21, 3.26)	0.39 (0.07, 1.86)	0.94 (0.18, 3.67)	0.65 (0.08, 3.55)
	**HFOV**	0.62 (0.18, 1.55)	0.29 (0.05, 0.92)	0.69 (0.14, 1.88)	0.48 (0.06, 1.89)
		**LPV**	0.47 (0.18, 1.03)	1.11 (0.54, 1.84)	0.78 (0.19, 2.32)
			**NMBA**	2.36 (0.79, 6.91)	1.66 (0.34, 7.03)
				**Open lung strategy using RM and/or higher PEEP**	0.70 (0.17, 2.61)
					**Prone positioning**

Each cell gives the effect of the column-defining intervention relative to the row-defining intervention.

HFOV: high frequency oscillatory ventilation, LPV: lung protective ventilation, NMBA: neuromuscular blockade, PEEP: positive end-expiratory pressure, RM: recruitment maneuver, VV ECMO: venovenous extracorporeal membrane oxygenation

© 2019 Aoyama H et al. *JAMA Network Open.*

eTable 11. Network Meta-analysis for Barotrauma and Quality of Evidence Assessment

Source	Risk of Bias	Heterogenity	Indirectness	Impresicion	Others	Direct Risk Ratio (95%CrI)	QOE	Indirect Risk Ratio (95%CrI)	QOE	Node splitting	Network Risk Ratio (95%CrI)	QOE
LPV (reference)				OIS=236								
ECLS	Not serious	Not serious	Not serious	Serious[c]	No	1.19 (0.24, 5.88)	Moderate	NA[f]	NA[f]	NA[g]	1.19 (0.24, 5.88)	Moderate
HFOV	Not serious	Serious[b]	Not serious	Serious[c]	No	1.69 (0.55, 7.14)	Low	NA[f]	NA[f]	NA[g]	1.69 (0.55, 7.14)	Low
NMBA	Not serious	Serious[b]	Not serious	Serious[c]	No	0.47 (0.18, 1.03)	Low	NA[f]	NA[f]	NA[g]	0.47 (0.18, 1.03)	Low
OPENLUNG	Not serious	Serious[b]	Not serious	Serious[c]	No	1.11 (0.54, 1.84)	Low	NA[f]	NA[f]	NA[g]	1.11 (0.54, 1.84)	Low
Prone	Not serious	Serious[b]	Not serious	Serious[c]	No	0.78 (0.19, 2.32)	Low	NA[f]	NA[f]	NA[g]	0.78 (0.19, 2.32)	Low
ECLS (reference)				OIS=218								
HFOV	Not serious	Serious[b]	Serious	Serious[c]	No	NA[f]	NA[f]	1.35 (0.21, 8.47)	Very low	NA[g]	1.35 (0.21, 8.47)	Very low
NMBA	Not serious	Serious[b]	Serious	Serious[c]	No	NA[f]	NA[f]	0.39 (0.07, 1.86)	Very low	NA[g]	0.39 (0.07, 1.86)	Very low
OPENLUNG	Not serious	Serious[b]	Serious	Serious[c]	No	NA[f]	NA[f]	0.94 (0.18, 3.67)	Very low	NA[g]	0.94 (0.18, 3.67)	Very low
Prone	Not serious	Serious[b]	Serious	Serious[c]	No	NA[f]	NA[f]	0.65 (0.08, 3.55)	Very low	NA[g]	0.65 (0.08, 3.55)	Very low
HFOV (reference)				OIS=214								
NMBA	Not serious	Serious[b]	Serious	Serious[c]	No	NA[f]	NA[f]	0.29 (0.05, 0.92)	Very low	NA[g]	0.29 (0.05, 0.92)	Very low
OPENLUNG	Not serious	Serious[b]	Serious	Serious[c]	No	NA[f]	NA[f]	0.69 (0.14, 1.88)	Very low	NA[g]	0.69 (0.14, 1.88)	Very low
Prone	Not serious	Serious[b]	Serious	Serious[c]	No	NA[f]	NA[f]	0.48 (0.06, 1.89)	Very low	NA[g]	0.48 (0.06, 1.89)	Very low
NMBA (reference)				OIS=243								
OPENLUNG	Not serious	Serious[b]	Serious	Serious[c]	No	NA[f]	NA[f]	2.36 (0.79, 6.91)	Very low	NA[g]	2.36 (0.79, 6.91)	Very low
Prone	Not serious	Serious[b]	Serious	Serious[c]	No	NA[f]	NA[f]	1.66 (0.34, 7.03)	Very low	NA[g]	1.66 (0.34, 7.03)	Very low
OPENLUNG (reference)				OIS=233								
Prone	Not serious	Serious[b]	Serious	Serious[c]	No	NA[f]	NA[f]	0.70 (0.17, 2.61)	Very low	NA[g]	0.70 (0.17, 2.61)	Very low

When there was no direct comparison, the quality of evidence of indirect comparisons was inferred from the lower quality of evidence among direct comparisons within the first-order loop.

a: The sensitivity analysis excluding studies with high risk of bias greatly changed the RR estimate

b: Moderate heterogeneity (I-squared = 30-60%) or substantial heterogeneity (I-squared = 60-90%) was found

c: The sample size did not reach the optimal information size (OIS)

d: The OIS met, but CrI included RR of 1.0

e: 95%CrI included benefit (RR=0.8) and harm (RR=1.25)

f: Not available due to no direct comparison or no indirect comparison

g: Not available, due to eithr no direct comaprison or no indirect estimate secaondary to lack of first closed loop

© 2019 Aoyama H et al. *JAMA Network Open.*

eTable 12. Results of Node-Splitting Models

Node splitting results: HFOV vs. LPV (reference)	
	**Median (95% CrI)**
**Direct estimate**	-0.130 (-0.448, 0.187)
**Indirect estimate**	0.023 (-1.316, 1.261)
**Inconsistency factor**	-0.148 (-1.419, 1.217)
**Inconsistency P-value**	0.823

Node splitting results: Prone positioning vs. LPV	
	**Median (95% CrI)**
**Direct estimate**	-0.396 (-0.764, -0.037)
**Indirect estimate**	-0.234 (-1.524, 0.956)
**Inconsistency factor**	-0.156 (-1.409, 1.191)
**Inconsistency P-value**	0.806

Node splitting results: HFOV vs. Prone positioning (reference)	
	**Median (95% CrI)**
**Direct estimate**	-0.342 (-1.639, 0.797)
**Indirect estimate**	-0.521 (-1.008, -0.031)
**Inconsistency factor**	0.168 (-1.193, 1.432)
**Inconsistency P-value**	0.798

A *P* value less than 0.05 indicated significant inconsistency between direct and indirect effect estimates.

HFOV: high frequency oscillatory ventilation, LPV: lung protective ventilation

© 2019 Aoyama H et al. *JAMA Network Open.*

eTable 13. Summary of Cointerventions in Included Studies

Trials	Intervention	Control	# of patient
HFOV			
Ferguson 2013	**During the First 28 Days of Study**<br>NMBA 82.9% / 3(1-6) days<br>Inhaled NO 9.1%<br>Prone positioning 2.6%<br>VV ECMO 1.1%	**During the First 28 Days of Study**<br>NMBA 68.1% / 2 (0-4) days<br>Inhaled NO 10.3%<br>Prone positioning 3.7%<br>VV ECMO 1.1%	548
Young 2013	NMBA used for 2.5 ± 3.5 days	NMBA used for 2.0 ± 3.4 days	795
Mohamed 2016	Not available	Not available	60
VV ECMO			
Peek 2009	HFOV 7%<br>Inhaled NO 10%<br>Prone positioning 4%	HFOV 14%<br>Inhaled NO 7%<br>Prone positioning 42%	180
Combes 2018	Prone positioning 66%<br>RM 22%<br>Inhaled NO (or prostacyclin) 60%	Prone poisoning 90%<br>RM 43%<br>Inhaled NO (or prostacyclin) 83%	249
Prone positioning			
Fernandez 2008	Not available	Not available	40
Taccone 2009	VV ECMO 1.1%	VV ECMO 1.1%	342
Guerin 2013	NMBA 91% (at inclusion)	NMBA 82.3% (at inclusion)	466
NMBA			
Gainnier 2004	Inhaled NO 10.7%<br>Prone positioning 14.3%	Inhaled NO 14.3%<br>Prone positioning 14.3%	56
Forel 2006	Inhaled NO 1pt<br>Prone positioning None	Inhaled NO 1pt<br>Prone positioning None	36
Papazian 2010	Prone positioning 28%<br>Inhaled NO 28%	Prone positioning 29%<br>Inhaled NO 33%	339
Guervilly 2017	Not available	Not available	24
PETAL 2019	**During the First 28 Days of Study**<br>Inhaled NO 1.4%<br>Prone positioning 16.8%<br>VV ECMO 0.6%<br>RM 5.8%	**During the First 28 Days of Study**<br>Inhaled NO 3.4%<br>Prone positioning 14.9%<br>VV ECMO 2.0%<br>RM 5.9%	1006
Open lung strategy using RM and/or higher PEEP			
Brower 2004<br>Meade 2008<br>Mercat 2008	Total use of rescue therapy in 3RCTs 13.7%	Total use of rescue therapy in 3RCTs 21.3%	2299
Talmor 2008	Not available	Not available	61
Huh 2009	Prone positioning 50%	Prone positioning 44.4%	57

© 2019 Aoyama H et al. *JAMA Network Open.*

	Inhaled NO 53.3%	Inhaled NO 48.1%	
Xi 2010	Not available	Not available	110
Hodgson 2011	None	Inhaled NO 1pt	20
Kacmarek 2016	Not available	Not available	200
Cavalcanti 2017	**During the First 7 Days of Treatment**<br>Prone positioning 15.2%<br>Inhaled NO 1.2%<br>HFOV 0.4%<br>VV ECMO 1.0%	**During the First 7 Days of Treatment**<br>Prone positioning 16.1%<br>Inhaled NO 1.2%<br>HFOV 0.6%<br>VV ECMO 1.0%	1010
Beitler 2018	Prone positioning 1.0%<br>Inhaled pulmonary vasodilator (e.g. inhaled NO) 2.9%<br>RM 1.0%<br>VV ECMO 1.0%	Prone positioning 3.1%<br>Inhaled pulmonary vasodilator (e.g. inhaled NO) 10.2%<br>RM 1.0%<br>VV ECMO 3.1%	200
Inhaled NO, Inhaled NOand RM			
Park 2003	Not available	Not available	23
HFOV and prone positioning			
Papazian 2005	Not available	Not available	39

HFOV: high frequency oscillatory ventilation, inhaled NO: inhaled nitric oxide, LPV: lung protective ventilation, NMBA: neuromuscular blockade, PEEP: positive end-expiratory pressure , RM: recruitment maneuver, VV ECMO: venovenous extracorporeal membrane oxygenation

© 2019 Aoyama H et al. *JAMA Network Open.*

eReferences

1. Higgins J, Green S, eds. *Cochrane Handbook for Systematic Reviews of Interventions Version 5.1.0*. The Cochrane Collaboration; 2011.
2. Salanti G. Indirect and mixed-treatment comparison, network, or multiple-treatments meta-analysis: many names, many benefits, many concerns for the next generation evidence synthesis tool. *Res Synth Methods*. 2012;3(2):80-97. doi:10.1002/jrsm.1037.
3. Spiegelhalter DJ, Best NG, Carlin BP, Van Der Linde A. Bayesian measures of model complexity and fit. *J R Stat Soc Ser B Stat Methodol*. 2002;64(4):583-616. doi:10.1111/1467-9868.00353.

© 2019 Aoyama H et al. *JAMA Network Open.*
